# Invasive Fishes Interact With Temperature to Reshape Community Size Structure Across Climatic Zones

**DOI:** 10.1111/gcb.70884

**Published:** 2026-04-27

**Authors:** Barbbara Silva Rocha, Ignasi Arranz, Henrique C. Giacomini, Daniel M. Perkins, Gilberto Nepomuceno Salvador, José Luís Costa Novaes, Jorge Iván Sánchez‐Botero, Angelo Antonio Agostinho, Paulo Santos Pompeu, Rosemberg Fernandes Menezes, Silvia Yasmin Lustosa‐Costa, Telton Pedro A. Ramos, José Luiz Attayde, Rodrigo Silva da Costa Goldbaum, Christine Argillier, Ronaldo César Gurgel‐Lourenço, Leonardo Mesquita Pinto, Tiago Casarim Pessali, María José Rodríguez‐Pérez, Victor Satoru Saito

**Affiliations:** ^1^ Environmental Sciences Department Federal University of São Carlos São Carlos São Paulo Brazil; ^2^ Instituto de Investigación en Cambio Global (IICG‐URJC) Universidad Rey Juan Carlos Móstoles España; ^3^ Departamento de Biología y Geología, Física y Química Inorgánica Universidad Rey Juan Carlos (URJC) Móstoles España; ^4^ Ontario Ministry of Natural Resources Aquatic Research and Monitoring Section Peterborough Ontario Canada; ^5^ Centre for Pollution Research and Policy Brunel University London Uxbridge UK; ^6^ Programa de Pós‐Graduação em Ecologia, Conservação e Manejo da Vida Silvestre Universidade Federal de Minas Gerais Belo Horizonte MG Brazil; ^7^ Biosciences Department Federal Rural University of the Semi‐Arid Mossoró Brazil; ^8^ Laboratório de Ecologia Aquática e Conservação (LEAC), Departamento de Biologia Universidade Federal Do Ceará – Campus Do Pici Fortaleza Brazil; ^9^ Programa de Pós‐Graduação Em Ecologia de Ambientes Aquáticos Continentais da Universidade Estadual de Maringá Maringá PR Brazil; ^10^ Departamento de Ecologia e Conservação Universidade Federal de Lavras, Campus Universitário Lavras MG Brazil; ^11^ Departamento de Fitotecnia e Ciências Ambientais, Centro de Ciências Agrárias Universidade Federal da Paraíba (UFPB) Areia PB Brazil; ^12^ Instituto Peixes da Caatinga João Pessoa PB Brazil; ^13^ Programa de Pós‐Graduação Em Sistemática e Evolução (UFRN), Departamento de Botânica e Zoologia, Centro de Biociências Universidade Federal do Rio Grande do Norte Natal RN Brazil; ^14^ Departamento de Ecologia, Centro de Biociências Universidade Federal do Rio Grande do Norte (UFRN) Natal Rio Grande do Norte Brazil; ^15^ Departamento de Biociências Universidade Federal Rural do Semi‐Árido Mossoró Rio Grande do Norte Brazil; ^16^ INRAE Aix Marseille University, UMR Recover Aix‐en‐Provence France; ^17^ Universidade Federal de Minas Gerais Belo Horizonte MG Brazil; ^18^ Confederación Hidrográfica del Ebro Paseo Sagasta Zaragoza Spain

**Keywords:** biomass, body size spectrum, freshwater communities, non‐native fish, trophic levels

## Abstract

The body size spectrum (or individual size distribution) is a simple yet widely recognized approach that links individual and population traits to community structure and ecosystem functions, making it a valuable indicator of anthropogenic effects. However, the assessment of size spectra in the context of biological invasions remains poorly explored. We investigated the impacts of non‐native (NN) fish invasions on the size structure of 667 lacustrine fish communities across climatic regions (temperate, tropical, and subtropical systems) and the roles of trophic position and temperature in modulating these effects. We found that fish communities under higher invasion pressure exhibit flatter, or less negative, size spectrum exponents. Also, NN species from lower trophic levels can have greater impacts than piscivorous NNs by reshaping size spectra and reducing the overall biomass of native communities. We also observed that piscivore NNs and NNs from lower trophic levels interacted positively with temperature to drive the size spectrum exponent and total biomass of the native communities, respectively. These results can be explained by two main mechanisms: (i) NN piscivores primarily act through size‐selective predation (top–down control), which may be intensified particularly on small prey in warmer lakes, and (ii) NN fish from lower trophic levels primarily act through competition, hence reducing the numerical abundance of small‐sized native fish, which may be more vulnerable in colder and less productive lakes. These mechanisms are leading to flatter size spectrum exponents mainly at higher temperatures and to a decline in the total biomass of the native community, mainly at lower temperatures, effectively reversing the expected temperature–size rule pattern. By disentangling the trophic and temperature‐dependent mechanisms through which NN fishes affect size structure, this study strengthens our ability to anticipate the impact of biological invasions on freshwater communities and their ecosystem functions and services under global change.

## Graphical abstract

Non‐native fishes are reshaping lacustrine communities across climatic zones worldwide through trophic‐ and temperature‐dependent pathways that are leading to flatter size spectrum exponents and a decline in the total biomass of the native community. Non‐native species from lower trophic levels exert stronger and more consistent effects than piscivorous invaders overall, flattening native size spectra and reducing biomass. In contrast, piscivorous invaders show stronger effects on size spectrum flattening with warming, whereas lower‐trophic invaders have stronger negative effects on native total biomass in colder systems. Together, these findings reveal the mechanisms by which biological invasions modify canonical temperature–size patterns in freshwater ecosystems.

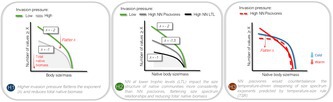

## 1 Introduction

Body size is a fundamental trait that governs the pace of life, influencing key biological processes such as metabolism, biomass production, lifespan, and feeding (Brose et al. 2006; Brown et al. 2004; Peters 1986). In body size studies, researchers have increasingly used the size spectrum, or individual size distribution (ISD), to describe how individuals are distributed across body sizes within a community (Edwards et al. 2017; Petchey and Belgrano 2010; White et al. 2007). The size spectrum generally follows a negative power scaling law, in which abundance declines systematically with body size (Marquet et al. 2005; Sheldon et al. 1972). This simple yet powerful approach provides a direct link between individual traits and community‐level patterns (White et al. 2007; Woodward et al. 2005). At the food web level, this scaling pattern provides a measure of energy flow from smaller to larger organisms (Silvert and Platt 1978). While the size spectrum exponent λ (i.e., analogous to slope) directly reflects size (or mass) distribution, the total biomass represents the standing stock and may serve as a proxy for the community's carrying capacity under stable conditions (Maury et al. 2007). Assessing these metrics related to body size structure serves as a valuable indicator of anthropogenic effects (dos Santos et al. 2017). In aquatic communities, for example, size spectrum relationships are sensitive to the impact of fishing (Fabré et al. 2017; Robinson et al. 2017), climate change (Queirós et al. 2018), eutrophication (Brucet et al. 2013), and land use alterations (Collyer et al. 2023).

In freshwater ecosystems, biological invasions are among the major threats to biodiversity (Ricciardi 2007; Simberloff et al. 2013), which can also modify community size structure (Arranz et al. 2023; Buba et al. 2017; Kopf et al. 2019; Moi et al. 2025). This is especially true for fish, which represent one of the most introduced groups in these systems (Gozlan et al. 2010). The detrimental impacts of invasive fishes on resident communities manifest through various mechanisms, including direct negative biotic interactions, i.e., predation and competition (Gozlan 2008; Gozlan et al. 2010). Alternatively, indirect interactions involve alterations in environmental conditions (e.g., turbidity, habitat degradation) and ecosystem functions (Crooks 2002). Among the observed impacts of biological invasions, changes in native abundance or biomass are particularly prominent (Gallardo et al. 2016). These alterations are often reflected in the size structure of fish communities, where invasive species have been associated with less negative size spectrum exponents (i.e., flatter slopes) and reduced native biomass (Arranz et al. 2023; Kopf et al. 2019). Flatter exponents were mainly linked to the tendency of successful non‐native (NN) fishes to be larger than native species (Blanchet et al. 2010; Liu et al. 2017). Also, these larger NN species feed upon (when piscivorous) and/or compete with smaller native fish for food and space in the invaded ecosystem, causing a decrease in native abundance (or biomass) (Arranz et al. 2021; Kopf et al. 2019). Such changes in size structure may indicate imbalances in energy flow when ecosystems deviate from steady‐state conditions, often caused by disturbances (Collyer et al. 2023; Guiet et al. 2016; Perkins et al. 2019).

Despite growing recognition that biological invasions are reconfiguring aquatic communities, important knowledge gaps remain regarding their disruptive effects and mechanisms. For instance, most studies have a limited geographic scope, often focusing on temperate (Arranz et al. 2023; Marin et al. 2023) or subtropical regions (Kopf et al. 2019; Moi et al. 2025), but rarely systematically comparing them. Also, little is known about whether invasions affect size structure in tropical areas, where high biodiversity and more complex food webs are observed (Fernando 1994; Kalff and Watson 1986; Thompson et al. 2012). This gap is due not only to the scarcity of empirical data from these often underrepresented systems in ecological monitoring programs (Elliott et al. 2019) but also to the lack of systematic cross‐regional studies that enable direct comparisons among systems and a broader understanding of invasion impacts. Therefore, integrating and assessing comparable data across regions is essential for gaining a macroecological perspective on how NN species alter community size structure. Additionally, studies often treat NNs as a homogeneous group, overlooking their different trophic roles (Arranz et al. 2021; Marin et al. 2023, 2025). This aspect limits our ability to understand the mechanisms by which invasions reconfigure size structure within native communities through distinct biotic interactions, i.e., predation and competition. Early theoretical perspectives argued that piscivores would exert the most pervasive and disruptive impacts due to their direct top–down control of prey populations and size structure (Moyle and Light 1996; Wellborn et al. 1996). However, recent empirical evidence suggests that some NN species occupying lower trophic levels are strong competitors that can also reshape size spectrum exponents and significantly reduce native biomass (Flood et al. 2024; Kopf et al. 2019; Murry et al. 2024; Novak et al. 2024). Taken together, these points highlight the importance of explicitly evaluating the relative contributions of different trophic strategies in shaping the size structure of fish communities under the invasion process.

Beyond these limitations, another important knowledge gap, which also emphasizes the need for broader cross‐latitude studies, concerns how invasive species interact with environmental gradients, particularly temperature, to shape size structure. Temperature is a major driver of metabolism and energy flow in natural systems, playing a key role in shaping community structure in ecosystems (Saito et al. 2021). Increasing temperatures along a thermal gradient are expected to produce steeper size spectrum exponents (Daufresne et al. 2009). These patterns arise because higher temperatures accelerate metabolic rates, favoring smaller‐bodied organisms with faster life cycles (the temperature‐size rule, or TSR, Daufresne et al. 2009; Verberk et al. 2021). This shift tends to steepen the size spectrum exponents, as communities become increasingly dominated by small individuals (Pomeranz et al. 2022). In addition, the higher metabolic demand with warming leads to increased energy dissipation across trophic levels, resulting in higher energy turnover rates and a lower total community biomass (Barneche et al. 2021; Barneche and Allen 2018). However, a recent study found that biological invasions can reverse the expected TSR (i.e., flatter size spectrum slopes with warming) probably due to the presence of NNs that are larger than natives and thrive in warmer contexts (Arranz et al. 2023).

Assessing how different trophic groups of NN species interact with temperature can reveal how distinct biotic interaction pathways, such as predation and competition mechanisms, drive departures from TSR expectations in body size structure patterns of native communities. For instance, in aquatic systems, predator–prey dynamics are particularly sensitive to temperature, as warming enhances feeding activity and metabolic demand (Cheng et al. 2017). This sensitivity is particularly evident in warmer environments, where the relationship between predator–prey mass ratios and the exponents of the size spectrum is stronger than in colder ones (Coghlan et al. 2022). Increased predation pressure has been suggested as a main driver undermining core TSR assumptions by intensifying top–down control (targeting smaller size classes) and resulting instead in flatter size spectrum slopes with warming in invaded freshwaters (Marin et al. 2025). In this context, NN piscivores may have more disruptive effects under warming. This occurs because NN species compensate for a higher metabolic demand by increasing their predator–prey mass ratio in response to warming, feeding down the food web and targeting smaller prey (Arim et al. 2007).

**FIGURE 1:**
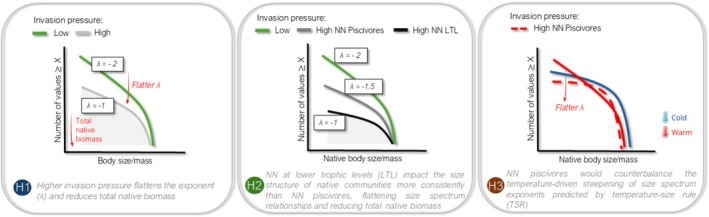
Conceptual framework illustrating the hypothesized effects of non‐native (NN) fish invasions on the size structure of freshwater fish communities. Each panel represents a distinct hypothesis (H1–H3) and presents stylized examples of individual size spectra (power‐law relationships) describing the distribution of fish body sizes under different invasion scenarios. These relationships are shown as linear trends on log–log axes, where the size spectrum exponent (*λ*) reflects the relative abundance of small versus large individuals. Values are used only to illustrate trends in *λ* and do not represent specific theoretical predictions. Changes in total native biomass are represented by the shaded grey area (i.e., the area under the curve). Together, the panels illustrate how variation in invasion pressure and the trophic identity of NN species is expected to alter both the exponent of the size spectrum and total native biomass across communities.

Because more energy is available in these smaller size classes, this response permits higher invasive biomass and flatter size spectrum slopes. In contrast, the role of NN species occupying lower trophic levels appears less directly influenced by temperature, as their competitive interaction effects also strongly depend on the availability of basal resources in ecosystems (Boets et al. 2019; George and Collins 2024). Being less contingent on temperature‐driven changes in metabolism and feeding behavior, these lower‐trophic NNs may exert stronger, more uniform effects on size structure across climatic zones worldwide. Therefore, understanding how distinct NN trophic groups interact with temperature is crucial for elucidating the mechanisms by which invasions alter size spectrum exponents and total biomass variation along environmental gradients, thereby leading to deviations from TSR predictions in ecological studies. In the context of accelerating global climate change, understanding these mechanisms may also help elucidate the effects of warming on community size structure, energy flow, and ecosystem functioning (Figure 1).

We aimed to investigate the impacts of NN fish invasions on the size structure of freshwater communities, both at the whole‐community level and specifically within native assemblages, across climatic zones. To assess the impacts of biotic interactions driven by NN species, we explored the mechanisms through which NN fishes occupying different trophic levels may affect native community size structure. We hypothesized that a higher invasion pressure flattens the exponent of the fish size spectrum and reduces native total biomass (H1). Although piscivores can strongly reshape community size structure by flattening the size spectrum, their effects are expected to be more sensitive to temperature (Coghlan et al. 2022). Therefore, we hypothesize that invasions by NN species occupying lower trophic levels impact the size structure of native communities more consistently than piscivorous invaders, flattening size spectrum relationships and reducing total biomass (H2). We also aim to infer how invasions by different trophic groups may disrupt the expected negative temperature–size relationships in natural freshwater communities. We hypothesized that NN piscivores would counterbalance the temperature‐driven steepening of size spectrum exponents predicted by TSR, as a higher metabolic demand in warmer environments favors a switch to smaller prey (H3) (Figure 1).

## 2 Methods

### 2.1 Biotic and Abiotic Data

**FIGURE 2:**
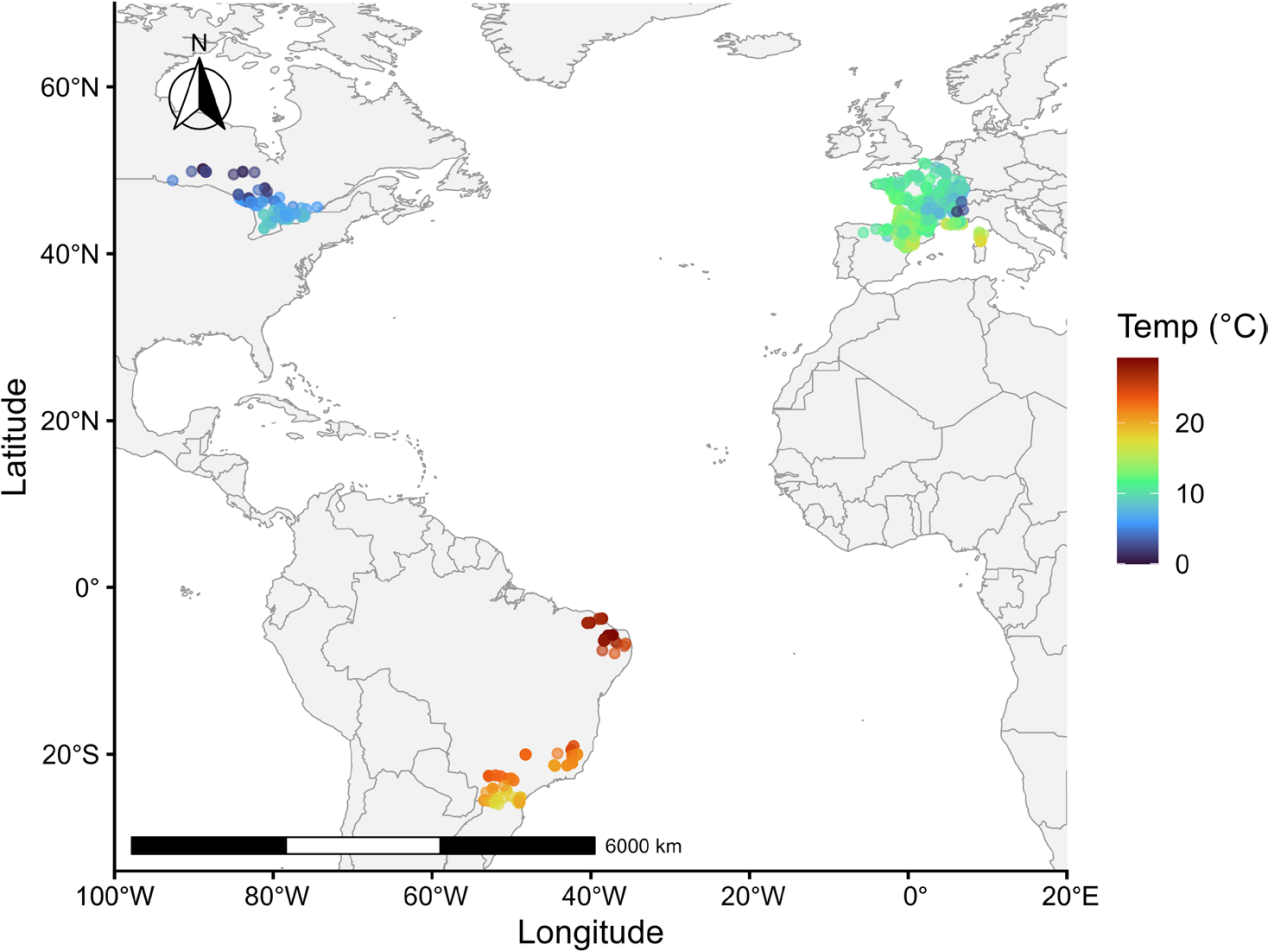
Map showing the 402 lakes (i.e., 120 natural and 282 artificial) sampled across temperate (Canada, France and Spain), subtropical and tropical (Brazil) climatic zones of the globe. Color represents the water temperature (“Temp.”) gradient. Map lines delineate study areas and do not necessarily depict accepted national boundaries.

We retrieved a unique database of individual fish body mass/size from multiple independent datasets sampled in natural and artificial (i.e., reservoir) lakes, between 2001 and 2024, encompassing temperate (Canada, France, and Spain) as well as subtropical and tropical (Brazil) climatic regions (Figure 2). Details on the data references for the sampling methodology of the different datasets are provided in Table S1. Fish surveys were conducted using benthic multimesh gillnets to ensure consistency and comparability in sampling effort and gear selectivity across all lakes. Fish caught were individually measured in total length (cm) and mass (g). For some individuals (0.01%) with missing body mass (g), we estimated mass using species‐specific length–weight relationships derived from the same dataset. For individuals missing both body mass and length (< 0.01%), we simulated body mass values using predictive modeling based on species identity and community size distributions (see Supporting Information). Only communities with at least two species (1 native and 1 NN) and 20 sampled individuals in total were selected. Including only communities where native and NN species coexist allows us to isolate and evaluate the effects of invasion pressure through their interactions, avoiding confounding factors that also affect size structure when comparing invaded and non‐invaded communities. Additionally, selecting a minimum number of sampled individuals ensures a reliable estimate of the size spectrum exponent by reducing bias associated with small sample sizes (Gerritsen and McGrath 2007). Some lakes had multiple sampling events, which ranged from 1 to 32 (mean = 2) per lake. In total, 636,980 individuals from 667 surveys across 402 lakes (120 natural and 282 artificial) were assessed.

We also collected information on abiotic factors known to affect the size structure of fish communities, including total phosphorus (μg/L; hereafter “TotP”), temperature (°C; hereafter “Temp”), and precipitation (Arranz et al. 2015, 2022; Emmrich et al. 2011; Rossberg et al. 2019). For TotP, we used the available measurements taken on the same or closest date to each fish sampling event. These measurements were taken throughout the water column at the deepest pelagic point of the lakes and were either provided by researchers or obtained from publicly available reports. The variable Temp. was represented by the mean annual air temperature (a proxy for water temperature) at the sampling point during the sampled year. In addition, we used both the absolute monthly precipitation for the month corresponding to the biological sampling and a relative measure calculated as monthly precipitation divided by the annual mean precipitation to capture both the magnitude and seasonal variation of precipitation rates. The relative measure allows differentiation between dry and wet periods within sites, independently of the total annual precipitation. We then performed a Principal Component Analysis (PCA) using both variables and extracted the first PCA axis (hereafter “Precip.”), which accounted for 87% of the variance among the lakes. This axis represents a gradient of precipitation intensity and seasonality, with higher values corresponding to lakes with higher precipitation levels and wet seasons. Temp. and Precip. data were obtained using the NASA Power API via the “nasapower” package in R (Sparks 2018). Additionally, we extracted information on lake area (in km^2^; “Lake_area”) and maximum depth (in m; “Max_depth”) by contacting data providers or retrieving data from available literature. Descriptive statistics for these variables across sites are provided in Table S2.

### 2.2 Fish Non‐Native Status and Trophic Guild

For each of the 404 fish species identified in surveys, we determined their status in each lake (native or NN), based on literature (Fricke 2021; Froese and Pauly 2024). A species was classified as non‐native if its occurrence was outside its native range. This category includes both exotic and translocated species. Translocated species are those whose native range includes freshwater ecoregions within the same country but not the specific ecoregion where the lake is located (Abell et al. 2008; Faria et al. 2025; Rocha et al. 2023; Su et al. 2019). We also determined the trophic guild of NN species (Froese and Pauly 2024), classifying them as piscivorous (exclusively fish predators when adults) or as members of lower trophic levels on the food web. These included omnivores (with a negligible piscivory diet), detritivores, herbivores, or invertivores (i.e., intermediate and basal trophic levels). We expected the piscivores group to mainly exert a predation effect on native species, particularly through the feeding behavior of adult individuals. Although juvenile piscivores are less likely to prey on other fish due to ontogenetic dietary shifts (Winemiller 1989), they can still impose significant nonconsumptive predator effects even when small (Tang et al. 2017). Such effects include fear responses triggered by predator‐associated cues in fishes (e.g., visual or chemical signals), which can reduce feeding, alter energy allocation, suppress reproductive activity, and ultimately affect metabolism and survival, regardless of the predator's size (Brown et al. 2011; Ferrari et al. 2010; Gjoni et al. 2025).

Therefore, our classification focuses on species‐level predator identity as a functional proxy for both consumptive and nonconsumptive effects of predator–prey interactions, rather than estimating realized piscivory at the individual level or by applying a size threshold, as used in previous research (Arranz et al. 2019; Mehner et al. 2016). Although omnivorous fish can consume other fish, they are classified into intermediate trophic guilds because their diet primarily consists of plant material, detritus, and low‐trophic‐level invertebrates, rather than a heavy reliance on piscivory (Gido and Franssen 2007). Therefore, omnivorous NN species, together with other non‐piscivorous species positioned at more basal trophic levels, are expected to exert competitive rather than predatory pressure on natives, primarily through the shared use of food, spawning sites, feeding habitats, or refuges.

To represent the overall fish invasion pressure, we measured the relative species richness (NN richness/total richness), relative abundance (NN abundance/total abundance), and relative biomass (NN biomass/total biomass) of NN species in each community. We performed a PCA analysis with these three variables following previous research (Catford et al. 2012), extracting the first principal component (“PCA_inv,” explaining 80% of the variance). More positive values along this axis indicate ecosystems that are highly dominated by NN species. We also determined the relative abundance of NN piscivorous species (“Pisc._NN”) and those from lower trophic levels (“LTL_NN”), calculated as the proportion of individuals from each group relative to the total fish community abundance, including both native and NN species. Dominance in this context reflects the increased probability and intensity of negative biotic interactions associated with each trophic group, allowing us to explore their potential effects on native community structure, i.e., predator–prey and competitive interactions. The low correlation between these trophic variables (*r* = 0.08) suggests that the impacts of different trophic groups on community structure are independent.

### 2.3 Size Structure Parameters

To represent the size structure of each fish community, we used the local size spectrum (sensu White et al. 2007). It was estimated using either the whole fish community (native + NN) or only the native species, to tease out the effects of invasions on the native community size structure. We applied the maximum likelihood estimation (MLE) method to estimate the exponent (λ) of the individual size distribution (ISD), which is analogous to the size spectrum slope (Bolker 2008; Edwards et al. 2017; Hilborn and Mangel 1997). This method assumes that abundance or frequency declines as a power function of size, here represented by body mass (g). More negative values of exponents indicate that the ecosystem supports a proportionally lower abundance of larger organisms. In contrast, less negative values suggest a relatively higher abundance of large‐bodied individuals. The size spectrum was fitted using the negLL.PLB function from the *sizeSpectra* package (Edwards 2019), which estimates λ by minimizing the negative log‐likelihood of a bounded power‐law distribution (PLB), as represented in the following equations:

(1) $$\ell \left(\lambda ;x\right)=-n*\ln\left(\frac{\left(\lambda +1\right)}{x_{\max}^{\left(\;\lambda +1\right)}-x_{\min}^{\left(\;\lambda +1\right)}\;}\right)-\lambda *\sum_{i=1}^{n}\ln\left(xᵢ\right),\text{for}\;\lambda \neq -1$$

(2) $$\ell \left(\lambda ;x\right)=n*\ln\left(\ln(x_{\max}\right)-\ln\left(x_{\min}\right))+\sum_{i=1}^{n}\ln\left(xᵢ\right),\text{for}\;\lambda =-1$$

Here, ℓ(*λ;xᵢ*) is the negative log‐likelihood function used to estimate the exponent of the individual size spectrum for each site (i); *xᵢ* represents the observed body mass of individual fish; *x*_min_ and *x*_max_ are the minimum and maximum body mass values of the size data for each community, respectively; *n* is the number of individuals sampled and included for analysis; and *λ* is the exponent of the bounded power‐law distribution (Perkins et al. 2019), which corresponds to the slope parameter describing the shape of the size spectrum.

To construct the size spectrum, we used fish body mass rather than body length because mass provides more information on energy content and directly reflects trophic roles, and potential impacts on energy transfer within the community (Edwards et al. 2017). Recent studies, conducted in temperate lakes using the “CEN” sampling protocol, often used a minimum size threshold of 4 g to construct fish size spectra (Marin et al. 2023; Mehner et al. 2016). However, this cutoff can be somewhat arbitrary when comparing regions with markedly different faunal compositions, such as those in tropical regions, where species tend to be smaller (Lindsey 1966), and lower thresholds may be more appropriate (Moi et al. 2025). To evaluate the sensitivity of exponent estimates to the choice of minimum body mass, we recalculated size spectra using cutoffs of 0.5 g (the minimum observed value), 1, 2, 3, and 4 g, and assessed concordance among exponent values using Spearman's rank correlations across the independent datasets used in the present study. Exponents were strongly correlated across thresholds, with deviations detected only in the European dataset sampled under the “CEN” protocol (Figure S1), consistent with previously documented size‐selectivity limitations (Mehner et al. 2016).

In our dataset, individuals weighing < 4 g represented 13% of all sampled fish and excluding them would disproportionately remove small‐bodied species, particularly in tropical assemblages. Therefore, we retained all individuals within the observed size range (0.5–64,962 g; mean = 329 g, SD = 584 g), spanning approximately five orders of magnitude in body mass. To further assess the robustness of model results to the inclusion of small‐bodied individuals, we conducted sensitivity analyses by refitting the statistical models using size‐spectrum exponents estimated with a 4 g minimum body mass cutoff, the threshold recommended for datasets collected under the European “CEN” protocol. We then compared the direction and magnitude of model coefficients with those obtained using the full‐size range. Finally, we estimated total biomass for each community, separately for the whole and native communities. To account for differences in sampling effort when using gillnets, i.e., total net area and sampling duration across surveys, total biomass was expressed as BPUE (Biomass Per Unit Effort) in kg/km^2^/h (Bounas et al. 2021; Brucet et al. 2013). Then, four metrics were calculated: “exponent(all),” “exponent(native),” “tot_biomass(all),” and “tot_biomass(native).”

### 2.4 Statistical Analyses

To test the first hypothesis (H1), we first built two linear mixed models (LMMs; Bolker 2015) using data from the whole community (native + NN species), one for each response variable: exponent(all) and tot_biomass(all). Fixed predictors included PCA_inv, Temp., and their interaction term. We also included TotP, Precip., lake area, and depth as covariates, as well as species richness (sp_rich), given its potential influence on freshwater size structure patterns (Pigot, Dee, Richardson, et al. 2025). Dataset type and lake type (natural vs. artificial) were included as fixed factors to account for methodological differences (including heterogeneity in gillnets' selectivity) and system‐specific characteristics. These were modeled as fixed effects, as they had few levels (5 and 2, respectively), and treating them as random effects led to unstable variance estimates and poor model performance (Bolker 2008; Gomes 2022). Finally, sites with multiple samples were treated as independent observations, with nonindependence accounted for by including lake_ID as a random effect.

To test H2, we built two additional LMM models using only the native community, with the response variables exponent(native) and tot_biomass(native). In these models, PCA_inv was replaced by Pisc_NN and LTL_NN, along with their interactions with Temp, as key predictors. The interactions in models were included to test the H3. All previously described covariates were retained, and Lake_ID was included as a random effect. However, residual diagnostics from the LMM fitted to the models with size spectrum exponents—exponent(all) and exponent(native)—revealed violations of model assumptions (Figures S2 and S3), particularly due to the presence of outliers and heavy‐tailed residuals. These models also exhibited convergence issues and a singular random‐effects structure, in which the random‐effects variance was estimated as zero. To address this, we refitted the exponent models using robust linear models (RLMs; Maronna and Yohai 2000), which extend standard linear models by down‐weighting the influence of extreme observations. In these RLMs, Lake_ID was excluded as a random effect, resulting in improved model fit and more reliable estimates under data heterogeneity (Koller 2016).

We used the lmer function from the *lmerTest* package (Kuznetsova et al. 2017) to run the LMMs. Additionally, the lmrob function, from the *robustbase* package (Maechler et al. 2021), was used for the RLMs. The *R* ^2^ values for the LMMs were calculated using the r2 function from the *performance* package (Lüdecke et al. 2019). Multicollinearity among fixed effects was assessed using the check_collinearity function, also from the *performance* package. Most predictors showed low collinearity (VIF < 4) across all models. In models assessing size spectrum exponents, temperature, and dataset type showed moderate to high collinearity, which is expected given the latitudinal structure of the sampling units and the nature of multilevel categorical variables. However, removing the dataset type from these models did not alter the estimates or significance of the main predictors, indicating that collinearity did not bias our main findings. Residual model diagnostics were performed using the *DHARMa* package (Hartig and Hartig 2017; Figures S2–, S5). All continuous variables were log(*x* + 1) transformed, except for LTL_NN and Pisc_NN, which were square‐root transformed. They were also scaled to ensure parameter estimates were comparable in units.

To gain additional insights into the potential mechanisms underlying the observed effects of invasive species, we explored whether different NN trophic groups disproportionately affected native size structure. Specifically, we tested whether native biomass distribution across size classes was more strongly associated with Pisc_NN than with LTL_NN. Although temperature was not included in this analysis, the goal was to explore whether these two trophic groups may differ in their size‐selective impacts on native communities. To achieve this, individual body mass values were log‐transformed and grouped into 10 size classes using logarithmically spaced, equal‐width bins that covered the full range of observed body sizes across the entire dataset. For each sampling event, we calculated the total biomass of native fish within each size class. To normalize the data, we applied a log(*x* + 1) transformation to the size‐class frequencies. Spearman's rank correlations were then computed using the rcorr function from the *Hmisc* package. All analyses were performed in R version 4.5.0 (R Core Team 2025).

## 3 Results

**FIGURE 3:**
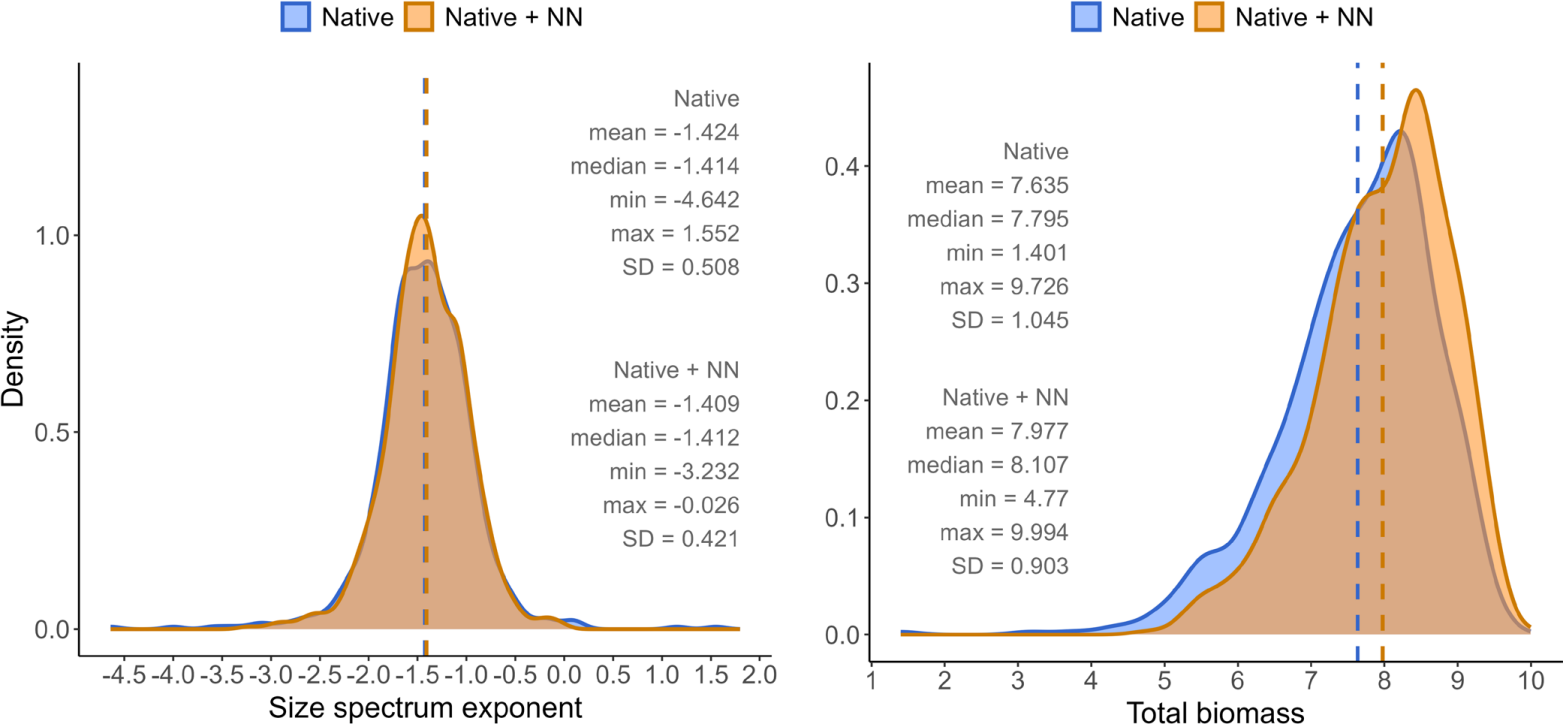
Density plots of size spectrum exponent (left panel) and log‐transformed total biomass (BPUE in kg/km^2^/h; right panel) for fish communities with native + NN species (orange) and only native species (blue). Dashed lines indicate means.

We found that size spectrum exponents (*λ*), extracted from the bounded power‐law distribution (see Figure S6 for representative rank–frequency plots), showed similar mean values between the two community compositions (Figure 2a). The size spectrum exponent values for the whole community (native + NN) ranged from −2.9 to −0.026 (mean = −1.25), while those for native‐only communities ranged from −3.7 to 1.55 (mean = −1.26). In contrast, we found a slight shift toward higher total biomass values when considering all species, both native and NN (mean = 7.97), compared to native‐only communities (mean = 7.63) (Figure 3).

### 3.1 Impacts of Invasion Pressure on Size Structure at the Whole‐Community Level

**FIGURE 4:**
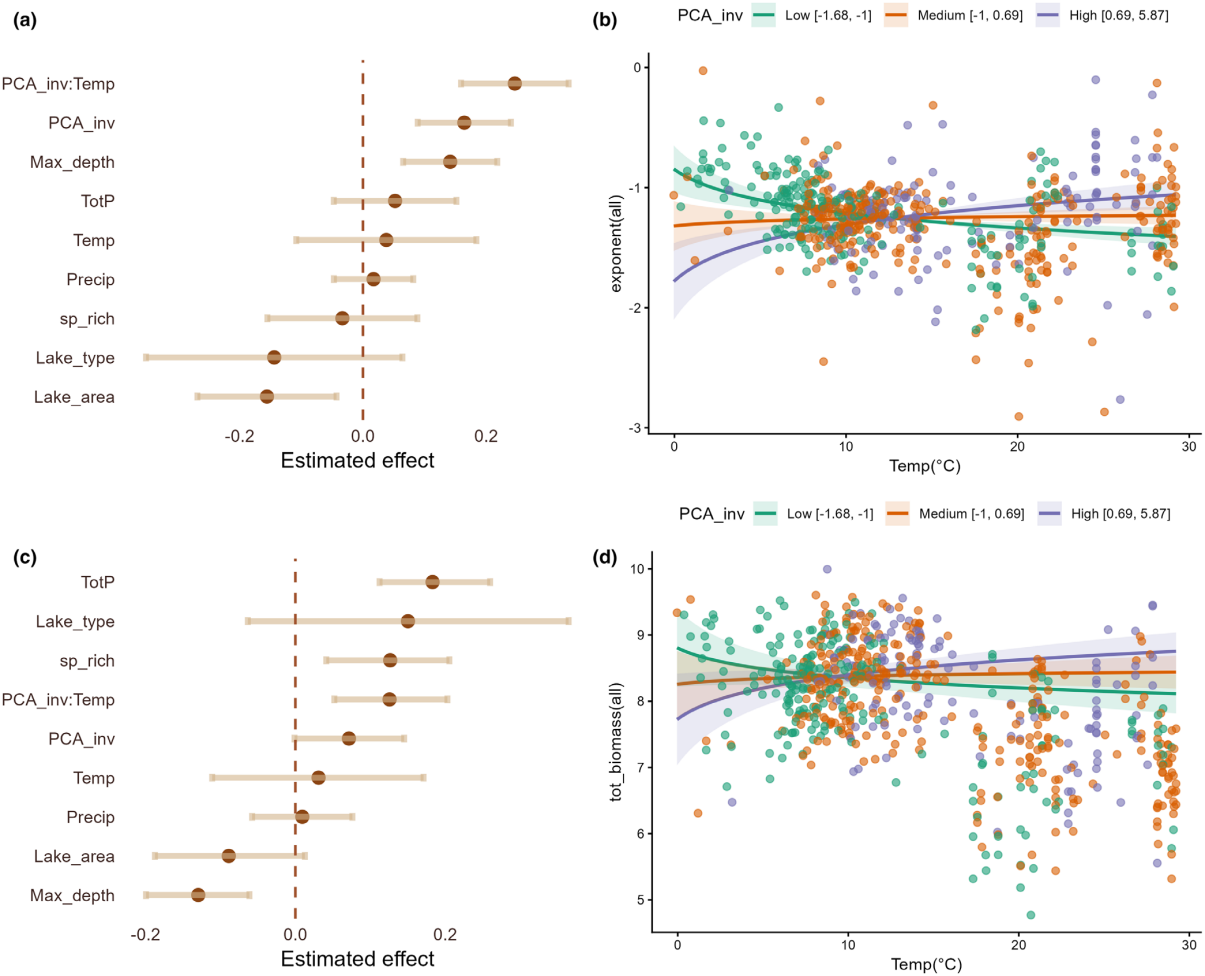
Effects of environmental and invasion pressure variables on size spectrum exponents and total biomass for the whole fish community (native + NN). Panel (a) shows the estimated coefficients (±95% CI) from robust RLM for the size spectrum exponent (exponent(all) model), and panel (b) depicts the interaction between invasion pressure (PCA_inv) and temperature (Temp). Effects (c) present the estimated coefficients from LMM for total biomass (tot_biomass(all) model), while panel (d) shows the interaction effect between PCA_inv and Temp on total biomass.

We found that communities with flatter size spectrum exponents were associated with greater invasion pressure in lake ecosystems. Specifically, PCA_inv showed a positive effect on the exponent(all) values (β = 0.164, *p* < 0.001), indicating that higher invasion pressure is associated with a shallower decline in organism abundance with body size (Figure 4a, Table S3). Although temperature alone did not significantly affect size spectrum exponent values, we observed a significant interaction between PCA_inv and Temp (β = 0.246, *p* < 0.001). This indicates that invasion pressure modulates the effect of the temperature gradient on the exponent. Specifically, we found that the TSR pattern, characterized by steeper exponents at higher temperatures, only emerged under low invasion pressure (Figure 4b). This pattern was inverted under high invasion pressure, leading to flatter size spectrum exponents in warmer conditions. Finally, the relationships between the covariates lake area (negative effect) and max_depth (positive effect) and the exponents were statistically significant (Figure 4a and Table S3). More specifically, larger lakes exhibited steeper size spectra, while deeper systems showed flatter exponents.

The interaction between Temp and PCA_inv also showed a significant positive relationship with tot_biomass(all) (β = 0.126, *p* = 0.001) (Figure 4c,d, Table S4). Biomass increased with temperature under high invasion pressure, while biomass decreased with temperature when invasion pressure was low. In summary, high invasion pressure led to flatter size spectrum exponents and greater total biomass in warmer lakes, contrasting with lower biomass and steeper size spectrum exponents in colder lakes. Regarding the covariates, sp_rich and TotP were positively associated with tot_biomass(all), whereas max_depth showed a negative relationship (Figure 4c and Table S4). In other words, total biomass increased with total phosphorus concentration and species richness but decreased with lake depth.

### 3.2 Impacts of NN Trophic Groups on Native Community Size Structure

**FIGURE 5:**
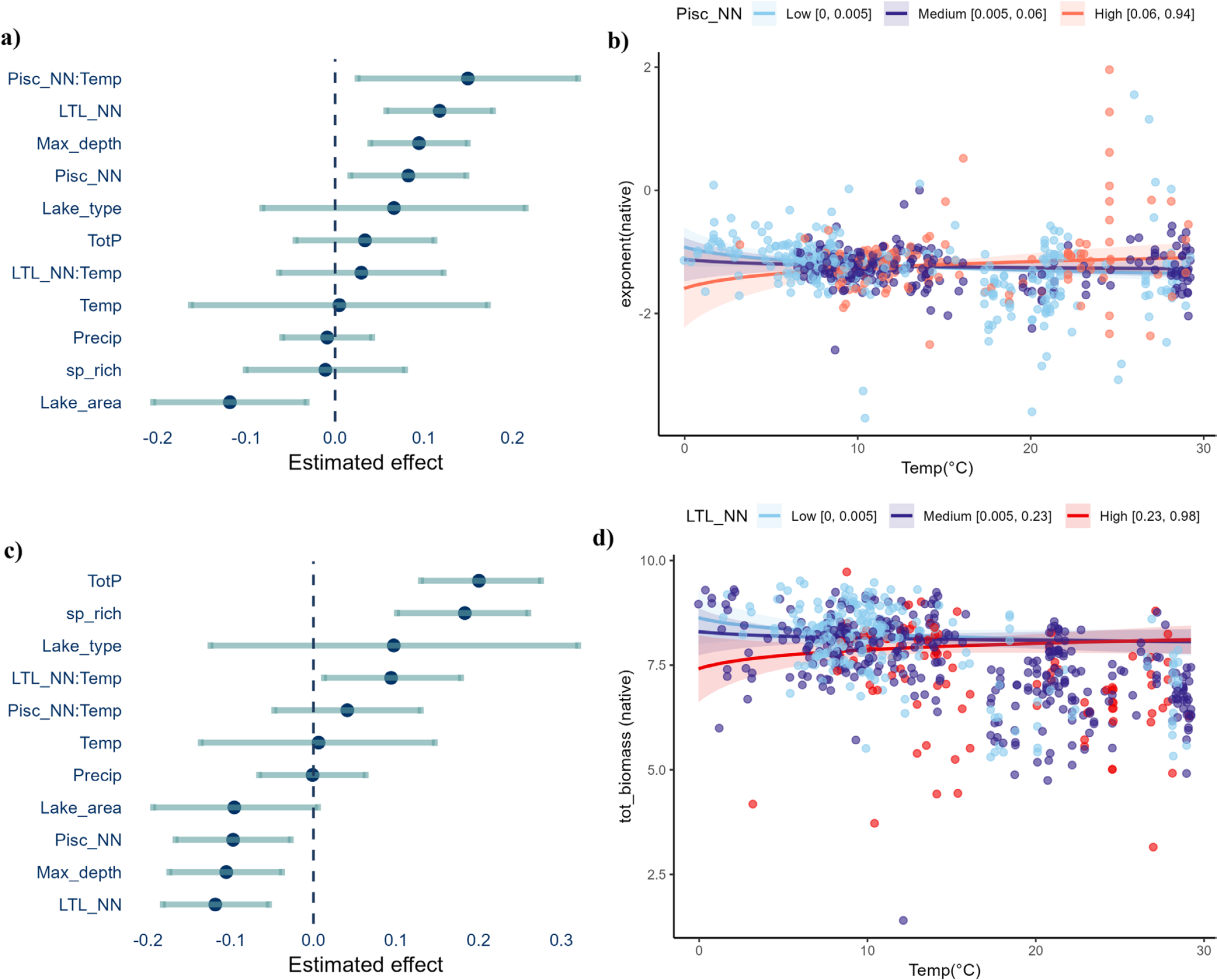
Effects of environmental variables and the dominance of different NN trophic groups on size spectra exponent and total biomass for the native fish community. Panel (a) shows the estimated coefficients (±95% CI) from RLM for the size spectrum exponent (exponent(native) model), and panel (b) depicts the interaction between NN piscivores (Pisc_NN) and temperature (Temp) affecting the exponent. Panel (c) presents the estimated coefficients from linear LMM for total biomass (tot_biomass(all) model), while panel (d) shows the interaction between other NN species occupying lower trophic levels in the food web (LTL_NN) and Temp affecting total biomass.

We found that both groups, piscivores and lower‐trophic‐level NNs, flattened the size spectrum exponents, with the latter showing a slightly stronger effect. More specifically, we found a positive relationship between exponent (native) values and the predictors Pisc_NN (β = 0.083, *p* = 0.013) and LTL_NN (β = 0.118, *p* < 0.001; Figure 5a and Table S5), indicating that the size spectrum exponent becomes less negative in local fish communities with high relative abundance of both NN trophic groups. Although Temp alone did not significantly affect exponents (β = 0.005, *p* = 0.955), we observed a significant positive interaction between Pisc_NN and Temp (β = 0.15, *p* = 0.018). This indicates that the pressure exerted by NN piscivores increases with temperature, with the size spectrum exponents becoming flatter at higher temperatures (Figure 5b and Table S5). The relationships between the covariates lake area (negative effect) and max_depth (positive effect) and the exponent values in native communities were statistically significant (Figure 5a and Table S5).

We also found that a high proportion of LTL_NN or Pisc_NN individuals reduced the total biomass of native communities in lakes, with LTL_NN having a slightly stronger effect. Specifically, LTL_NN (β = −0.119, *p* < 0.001) and Pisc_NN (β = −0.097, *p* = 0.007) were negatively related to tot_biomass values in lakes (Figure 5c and Table S6). Moreover, we found a significant positive interaction between LTL_NN and Temp (β = 0.094, *p* = 0.027) in the tot_biomass model (Figure 5d). Therefore, both NN groups had significant effects on the size spectrum exponent (positive) and total biomass (negative). However, Pisc_NN only interacted with temperature in the exponent model, whereas LTL_NN only interacted with temperature in the biomass model (Figure 5c and Table S6). Regarding the covariates, sp_rich and TotP were positively associated with tot_biomass (all), whereas max_depth showed a negative relationship (Figure 5c and Table S6). We highlight that sensitivity analyses using a 4 g minimum size cutoff yielded qualitatively similar parameter estimates and inferences, with no changes in the direction or statistical significance of key effects across all models with size spectrum exponents (Tables S7 and S8).

**FIGURE 6:**
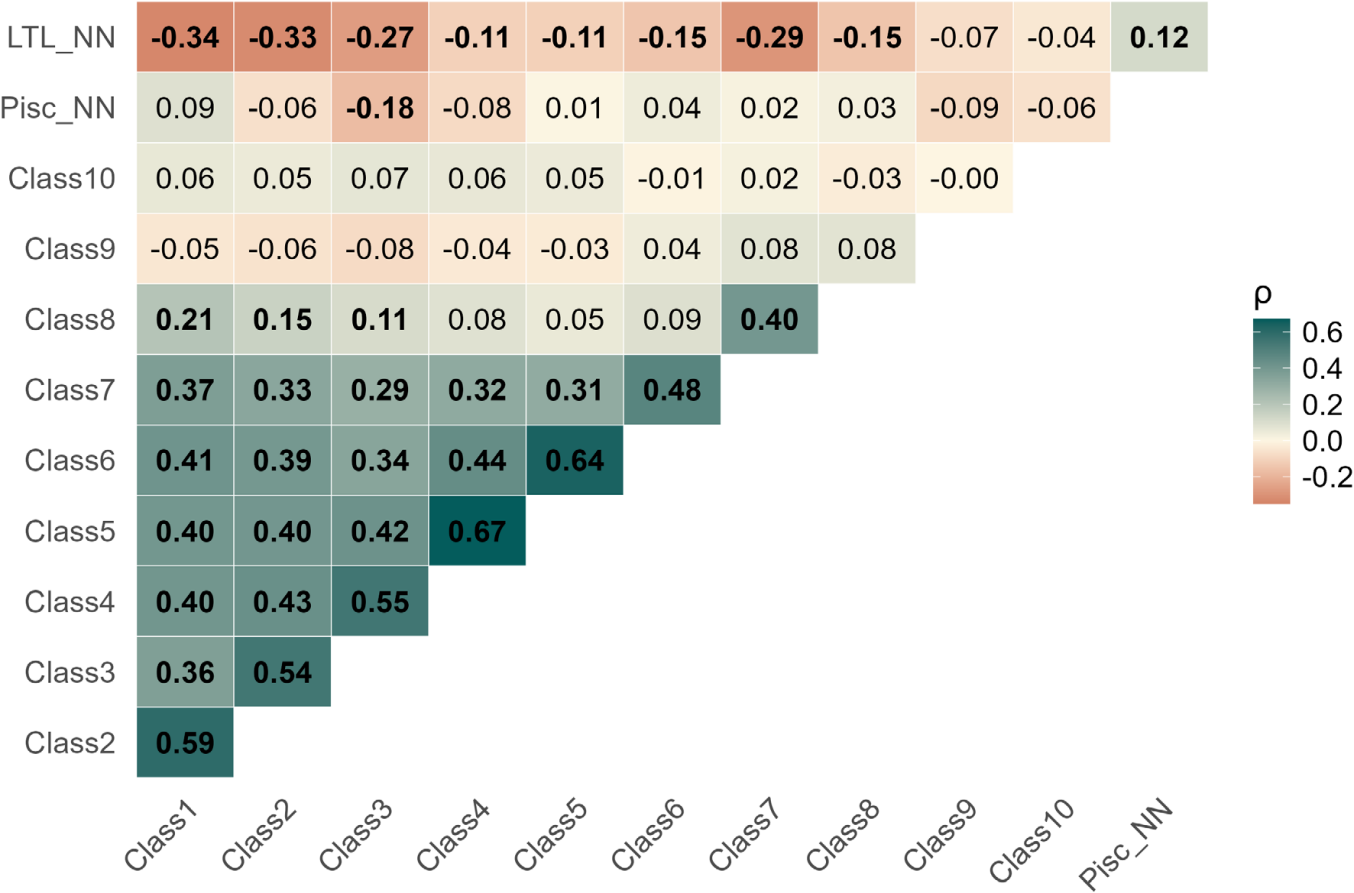
Spearman's correlation values (*ρ*) between dominance by the NN trophic groups (LTL_NN and Pisc_NN) and the total biomass of each 10 size class in native communities. The size classes were: Class1 [0.5–1.49 g]; Class2 [1.49–4.42 g]; Class3 [4.42–13.14 g]; Class4 [13.14–39.08 g]; Class5 [39.08–116.19 g]; Class6 [116.19–345.47 g]; Class7 [345.47–1027.18 g]; Class8 [1027.18–3054.11 g]; Class9 [3054.11–9080.8 g]; and Class10 [9080.8–64,962 g]. Values in bold are statistically significant (*p* < 0.05).

**FIGURE 7:**
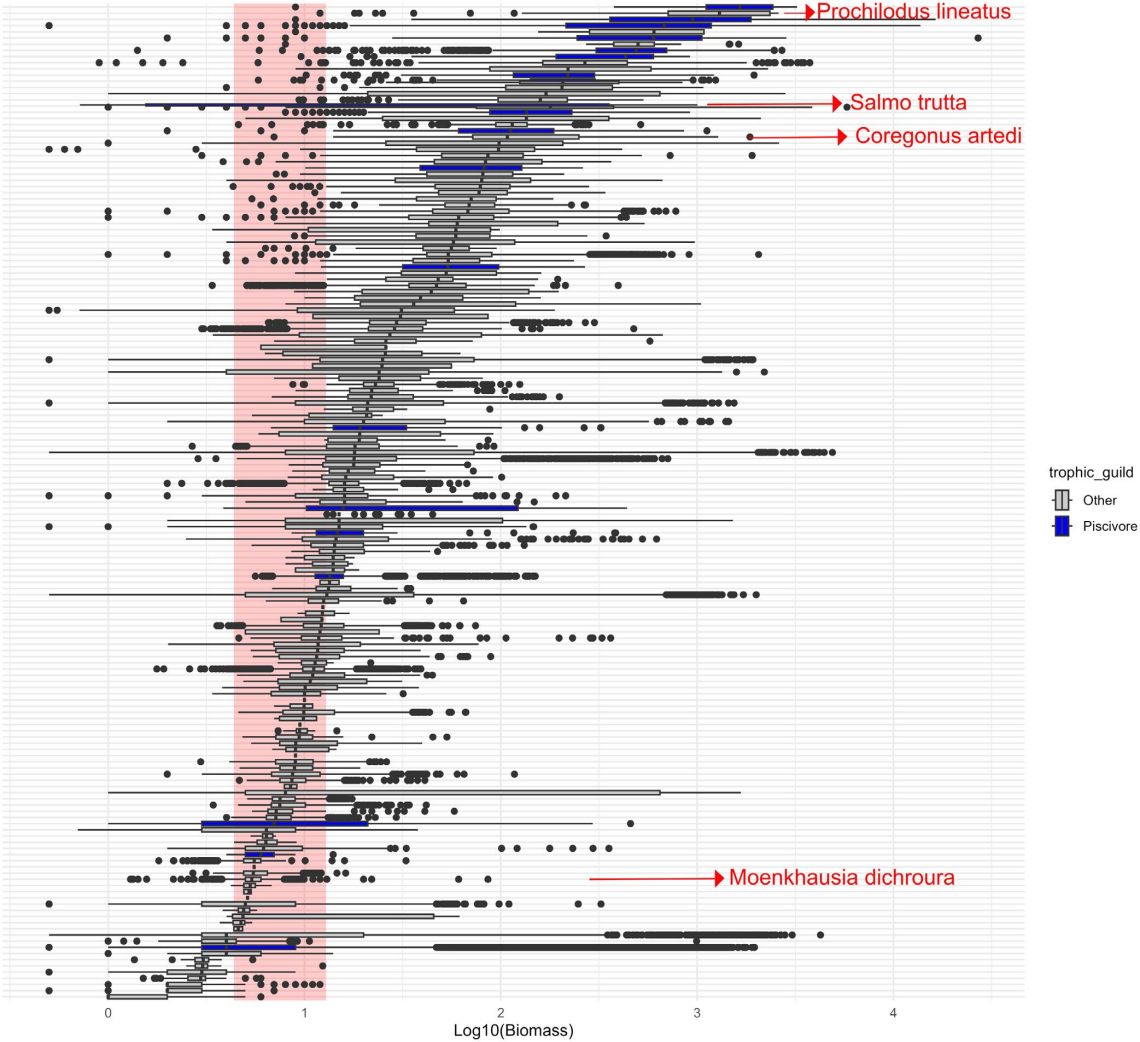
Distribution of individual body mass (log_10_‐transformed) for the 106 species occurring within their native range that fall within the size interval of Class3 [4.42–13.14 g] of the native community (highlighted by a red vertical band). This size class was negatively correlated with both NN trophic groups. Boxplot colors indicate the trophic guild of the affected native species (blue = piscivores; grey = other trophic groups). Species shown in red are examples cited in the discussion section, while the full list of species is provided in Figure S7.

Finally, a single significant negative correlation was found between Pisc_NN and the biomass of a smaller size class, specifically Class3 (*ρ* = −0.18) of natives, suggesting a specific size target is most impacted by this group (Figure 6). In contrast, the LTL_NN group showed multiple significant negative correlations across a broader range of native size classes. However, the strongest negative correlations were observed for the smaller classes: Class1 (*ρ* = −0.34), Class2 (*ρ* = −0.33), Class3 (*ρ* = −0.27), with Class7 (*ρ* = −0.29) as an exception (Figure 6). The Class3, ranging from 4.42 to 13.14 g, was negatively related to both NN groups and includes not only many small‐bodied species from both tropical and temperate regions, but also the juvenile stages of larger native fishes, which mainly occupy lower trophic levels in food webs (Figure 7).

## 4 Discussion

In this study, we aimed to understand how NN fish invasions affect the size structure of freshwater fish communities across broad geographic and environmental gradients, with a particular focus on the role of different NN trophic groups. We showed that NN fish invasions consistently reshape the size structure of freshwater communities, with their effects interacting with the temperature gradient across different climatic zones. Both NN groups, i.e., piscivores and especially lower‐trophic‐level species, contributed to this reshaping by flattening the exponents of the native size spectrum and reducing native biomass. On one hand, the effects of piscivorous invaders interacted with the temperature gradient in driving changes in size spectrum exponents. On the other hand, NN species at lower trophic levels interacted with temperature exclusively in driving total biomass. This suggests that distinct mechanisms of trophic groups modulate different components of community size structure under warming conditions.

Interestingly, invasion pressure did not influence community size‐spectrum exponents or total biomass in the same way across regions; rather, it interacted with thermal conditions. More specifically, in highly invaded ecosystems, warming conditions led to flatter exponents and greater total community biomass. This finding likely reflects the predominance of NN species with larger body sizes, greater thermal tolerance, and metabolic efficiency (Hutchings et al. 2025; Kłosiński et al. 2025). These traits enable them to maintain high growth rates and accumulate more biomass under elevated temperatures, particularly at the upper end of the community size spectrum, thereby flattening the size spectrum exponent and increasing total community biomass. This result contributes to a growing debate in the literature regarding temperature‐driven changes in size spectra (Gjoni et al. 2024), suggesting that interactions with global change factors, such as biological invasions, may help explain the lack of consensus by altering community responses to natural environmental gradients. Indeed, the effect of invasions on the reconfiguration of the temperature–size structure relationship (TSR) has already been highlighted in a recent study in French freshwater systems (Arranz et al. 2023). However, as expected, flatter exponents under warming conditions (the inverse of the TSR expectation) were observed only when the NN piscivore group was highly dominant. This is likely because NN piscivores often come from warmer regions or exhibit broader thermal performance curves, enabling them to sustain high metabolic activity and predation rates at high temperatures (Emiroğlu et al. 2023; Gkenas et al. 2022). Also, the NN piscivores tend to be highly selective toward small‐bodied native individuals, which are both more abundant and energetically profitable in warmer lakes (Emiroğlu et al. 2023). By contrast, piscivores in colder lakes, e.g., in temperate regions, exhibit lower metabolic rates and feeding activity, preferring to prey on a few, larger individuals (Bihun et al. 2024; Esin et al. 2021). Therefore, predator–prey interactions promoted by NN piscivores seem to play an essential role in reducing biomass accumulation in smaller size classes and flattening the native size spectrum exponent in warmer ecosystems compared to colder ones. This finding helps to corroborate our H3 that NN piscivores would counterbalance the temperature‐driven steepening of size spectrum exponents predicted by TSR, as higher metabolic demand in warmer environments favors a switch to smaller prey.

In turn, NN species at lower trophic levels inverted the expected decline in native total biomass under warmer conditions, despite not altering the TSR‐predicted steepening of size spectrum exponents. More specifically, under a high invasion scenario, fish communities in colder lakes sustained lower native total biomass than those in warmer lakes. This pattern can be explained by potentially intensified competitive impacts in colder environments, where primary productivity tends to be lower, and resources are more limited (McMeans et al. 2016; Salmaso et al. 2012; Shuter et al. 2012). In addition, native species in colder lakes often exhibit larger body sizes (Brucet et al. 2013) and are characterized by slower metabolism and delayed reproduction (K‐strategists), which may make their populations less resilient to exclusion by highly efficient NN competitors that can thrive even under resource‐limited conditions. Consequently, the loss of these large‐bodied native species may represent a disproportionate reduction in the potential for biomass accumulation in colder ecosystems.

Although dominance by these NN from lower trophic levels was associated with reduced native biomass, this effect appears to be less pronounced in warmer environments. In warmer ecosystems such as the tropics, native species at lower trophic levels tend to be smaller‐bodied (Lindsey 1966). Furthermore, warmer environments present high turnover of basal resources (Sarmento 2012), which can sustain higher trophic levels even when standing stocks are low, while the greater resource diversity allows NN generalists to exploit resources otherwise unused by natives. This potentially reduces direct NN–native competition and is consistent with Rocha and Cianciaruso (2020), who found that warmer lakes are associated with low competitive interactions, facilitating coexistence between NN and native communities. This specific effect of non‐piscivorous species on total biomass patterns is ecologically relevant, as total biomass can reflect a community's ability to store energy and contribute to ecosystem stability (Tilman 1996). Regarding the specific role of NN groups in interacting with temperature to drive patterns in the exponent or total biomass, we emphasize the heightened vulnerability of warmer environments, such as tropical systems, to NN piscivore‐driven shifts in community size structure. In contrast, colder systems (with lower resource availability) appear more susceptible to greater impacts of biomass losses promoted by NN species from lower trophic levels. These differences are likely driven by metabolism‐related life‐history traits of NN and native species, providing a unified mechanistic explanation for the contrasting results observed across systems at different locations. We highlight that experimental and theoretical frameworks tend to support a universal TSR trend, whereas empirical patterns in natural communities depend strongly on biotic context, including species interactions, trophic structure, and invasion dynamics. Finally, we suggest this could inform invasive management and policy, with actions focused on minimizing the impacts of target NN trophic groups on native biodiversity across different climates.

Overall, we found that biological invasions lead to an increase in the number of large‐bodied individuals when considering the whole community (native + NN species). This pattern likely results from two opposing but simultaneous mechanisms: an increase in total biomass driven by the presence of larger‐bodied NN species, and a reduction or replacement of native biomass due to negative biotic interactions. Indeed, previous studies have shown that successfully established NN fishes in freshwater systems worldwide tend to be larger than native species (Blanchet et al. 2010), especially those introduced for angling purposes (Su et al. 2019). It is possible that these species promote a top‐heavy trophic pyramid by shifting biomass toward upper trophic levels (Kopf et al. 2019). In parallel, invasions can often suppress native populations, mainly the smaller individuals, through direct and indirect biotic interactions (Fanson et al. 2024; Gozlan 2008; Gozlan et al. 2010). As a result, invasive fish species can dynamically redistribute biomass toward larger size classes across the whole community, thereby flattening the size spectrum slopes. This structural change would reflect a shift in how energy is stored and transferred within invaded lake communities. This is concerning, as altered size structure may reduce community stability and resilience, increasing the likelihood of cascading effects under further disturbances (Flood et al. 2024; McCann 2000; Novak et al. 2024). The role of NN species in altering the exponents of the fish size spectrum through these mechanisms is consistent with recent findings from subtropical (Moi et al. 2025) and temperate (Arranz et al. 2021, 2023; Novak et al. 2024) ecosystems. In our study, encompassing a broad temperature gradient, we provided empirical evidence that NN species systematically reshape the size structure of aquatic communities across different regions, including those in tropical areas.

We found that both groups, NN piscivores and those from lower trophic levels, contributed to the flattening of size spectra and the reduction in total biomass in native communities. More specifically, the potential predation pressure exerted by NN piscivores appears to primarily target smaller‐bodied native individuals occupying the smaller size fractions of the native size structure (Emiroğlu et al. 2023; Gratwicke and Marshall 2001; Pelicice et al. 2015). This may occur through direct biomass consumption, reflecting a strong size‐selective foraging behavior, and through indirect nonconsumptive effects such as behavioral changes induced by predation risk that negatively influence smaller prey (Godinho and Ferreira 2000; Tang et al. 2017). Likewise, competitive interactions likely imposed by NN species from lower trophic levels can also disproportionately impact smaller native individuals, which often correspond to early life stages or more specialized taxa that typically have lower foraging efficiency and competitive ability when compared to more generalist or opportunistic NNs' strategies (Faria et al. 2025). The above‐cited findings corroborate our first hypothesis (H1) that higher invasion pressure flattens the slopes of community size spectra and reduces native total biomass.

Interestingly, the slightly stronger effects on exponent flattening and native biomass reduction observed for lower‐trophic‐level NNs underscore the ecological importance of non‐piscivorous species. This pattern is consistent with previous studies on the pervasive effects of widespread detritivorous (e.g., armored catfish, Murry et al. 2024), planktivorous (e.g., *Hypophthalmichthys molitrix* , Novak et al. 2024), and omnivorous (e.g., *Cyprinus carpio* ; Kopf et al. 2019) NN species in flattening size distribution patterns or reducing native fish biomass across freshwater ecosystems worldwide. Therefore, we also corroborate our H2 that invasions by NN species occupying lower trophic levels can negatively impact size structure in native communities, flattening the size‐spectrum exponent and reducing total biomass more consistently than top‐predator invaders. Although piscivorous invaders strongly affect native populations through predator–prey interactions and top–down control (Moyle and Light 1996; Wellborn et al. 1996), NNs occupying lower levels in food webs can exert more substantial impacts. This is likely because they can broadly deplete resources (food and habitat) used by smaller size classes, ultimately leading to persistent reductions in native biomass and flatter size spectra.

We also observed that greater dominance of both NN groups was associated with a lower frequency of native individuals within a narrow body size range (4–13 g), suggesting that this size class may be particularly sensitive to changes in community composition. Within this, approximately 10 g of size variation is included in 106 species in our dataset, corresponding to 26% of all sampled fish species. This encompasses not only small native species with high endemism (e.g., *Moenkausia dichroura*), but more importantly, also juveniles of species with ecological and commercial interest, such as trout ( *Salmo trutta* ) and cisco ( *Coregonus artedi* ) in temperate/boreal lakes, and curimbatá ( *Prochilodus lineatus* ) in tropical systems. This potential pressure on smaller size classes of natives suggests that the primary mechanism for flattening the size spectrum exponents and reducing total biomass may not be the immediate loss of large‐bodied individuals or species, which would truncate the upper end of the size spectrum. The concern actually lies in how this mechanism may affect the most sensitive life stages (i.e., juveniles) of these species, which fall within the main impacted size range at the base of the native size spectrum. Therefore, biological invasions appear to act as a potential functional filter operating within a critical size window, generating an ecological exclusion zone that could disproportionately suppress small‐bodied species and early life stages. While the intensity of this size‐selective filtering likely varies across regions according to local environmental and biotic contexts, our findings suggest a general trend toward community size homogenization and a potential disruption of recruitment processes, even among larger‐bodied taxa. Such alterations can cascade through food webs, affecting energy transfer and ultimately compromising long‐term ecosystem functioning. Future studies should empirically test these potential mechanisms across broader ecological and geographical contexts.

In addition to invasion‐related predictors, we detected consistent effects of several environmental covariates on size spectrum patterns. Species richness was positively associated with both size spectrum exponent and biomass, consistent with the idea that more diverse assemblages sustain higher community biomass. This finding aligns with Pigot, Dee, Richardson, et al. (2025), who demonstrated that richness–biomass relationships in nature follow predictable macroecological rules, particularly in fish communities, given their wide variation in body size. Lake area showed a negative relationship with size spectrum exponent, suggesting that larger systems harbor steeper size spectra, possibly reflecting differences in resource distribution, habitat complexity, and the increased availability of refuges for smaller individuals (Jinks et al. 2019). In contrast, maximum depth showed a positive association with exponent patterns, consistent with the expectation that deeper lakes can support larger‐bodied individuals, flattening the size spectra (Emmrich et al. 2011). Finally, total phosphorus was positively associated with biomass, consistent with the well‐known role of nutrient availability in enhancing productivity (Yurk and Ney 1989). Although these environmental factors were not the central focus of our study, their significant contributions underscore the importance of considering diversity and habitat features when assessing the impacts of NN species (a biotic driver) on community size structure. Nevertheless, we are aware of some limitations in the study, such as not explicitly considering the invasion stage or other anthropogenic pressures beyond eutrophication, including land‐use changes and fishing pressure. Future studies should incorporate these aspects to advance our understanding of the specific ecological effects of the invasion process.

## 5 Conclusions

Our findings underscore the importance of considering not only invasion pressure but also the trophic identity of NN species when assessing impacts on community structure. Understanding the distinct mechanisms through which these groups operate is essential for predicting invasion outcomes under varying environmental conditions. Ongoing climate change is expected to amplify these effects, potentially intensifying pressure on native populations by increasing the energetic demands of organisms. From an ecological and conservation perspective, shifts in community size structure can reverberate through ecosystems, influencing energy flow, trophic dynamics, and emergent properties such as stability and resilience. Anticipating these changes is critical for safeguarding biodiversity and maintaining the functions and services provided by freshwater ecosystems under the growing pressure of biological invasions, a primary global driver of biodiversity change.

## Author Contributions

**Jorge Iván Sánchez‐Botero:** writing – review and editing, data curation. **José Luís Costa Novaes:** writing – review and editing, data curation. **Barbbara Silva Rocha:** conceptualization, methodology, writing – review and editing, formal analysis, data curation, writing – original draft. **Paulo Santos Pompeu:** writing – review and editing, data curation. **Rosemberg Fernandes Menezes:** data curation, writing – review and editing. **Angelo Antonio Agostinho:** writing – review and editing, data curation. **Gilberto Nepomuceno Salvador:** data curation, writing – review and editing. **Ignasi Arranz:** writing – review and editing, data curation. **Rodrigo Silva da Costa Goldbaum:** writing – review and editing, data curation. **Henrique C. Giacomini:** writing – review and editing, data curation. **Daniel M. Perkins:** writing – review and editing. **Leonardo Mesquita Pinto:** data curation, writing – review and editing. **José Luiz Attayde:** writing – review and editing, data curation. **Ronaldo César Gurgel‐Lourenço:** data curation, writing – review and editing. **María José Rodríguez‐Pérez:** data curation. **Tiago Casarim Pessali:** data curation, writing – review and editing. **Telton Pedro A. Ramos:** writing – review and editing, data curation. **Victor Satoru Saito:** conceptualization, methodology, formal analysis, writing – review and editing, supervision, writing – original draft. **Silvia Yasmin Lustosa‐Costa:** data curation, writing – review and editing. **Christine Argillier:** writing – review and editing, data curation.

## Conflicts of Interest

The authors declare no conflicts of interest.

## Supporting information


**Figure S1:** LMM Model's diagnostic for the exponent of the whole community.

**Figure S2:** LMM Model's diagnostic for total biomass of the whole community.

**Figure S3:** LMM Model's diagnostic for the exponent of the native community.

**Figure S4:** LMM Model's diagnostic for total biomass of the native community.

**Figure S5:** Rank–frequency plots illustrating the bounded power‐law distribution of the size spectrum that was built.

**Figure S6:** Distribution of individual body biomass of native species within the size Class3 [4.42–13.14 g].

**Figure S7:** Distribution of individual body biomass (log10‐transformed) for the 106 species occurring within their native range that fall within the size interval of Class 3 [4.42–13.14 g] of the native community (highlighted in red). This size class was found to be negatively correlated with both non‐native trophic groups. Boxplot colors indicate the trophic guild of the affected native species (blue = piscivores; grey = other trophic groups).

**Table S1:** Overview of the datasets.
**Table S2:** Descriptive statistics of abiotic and biotic covariates used in models.
**Table S3:** Results of Robust linear model (RLM) for the exponent of whole community (native + NN).
**Table S4:** Results of linear mixed model (LMM) for the total biomass of the whole community.
**Table S5:** Results of RLM for the slope of native community.
**Table S6:** Results of LMM for the total biomass of native community.
**Table S7:** Results ofRLM for the exponent of whole community (native + NN) using the cutoff of 4 g for the individual body weight.
**Table S8:** Results of RLM for slope of native community using the cutoff of 4 g for the individual body weight.

## Data Availability

The data and R code for the main statistical analyses supporting the findings of this study are available at https://zenodo.org/records/19560923.
