# Supplementary figures and images for: Invasive Fishes Interact With Temperature to Reshape Community Size Structure Across Climatic Zones
Source: Glob Chang Biol. 2026 Apr 27;32:e70884. doi: 10.1111/gcb.70884 (PMC13112343; doi:10.1111/gcb.70884)

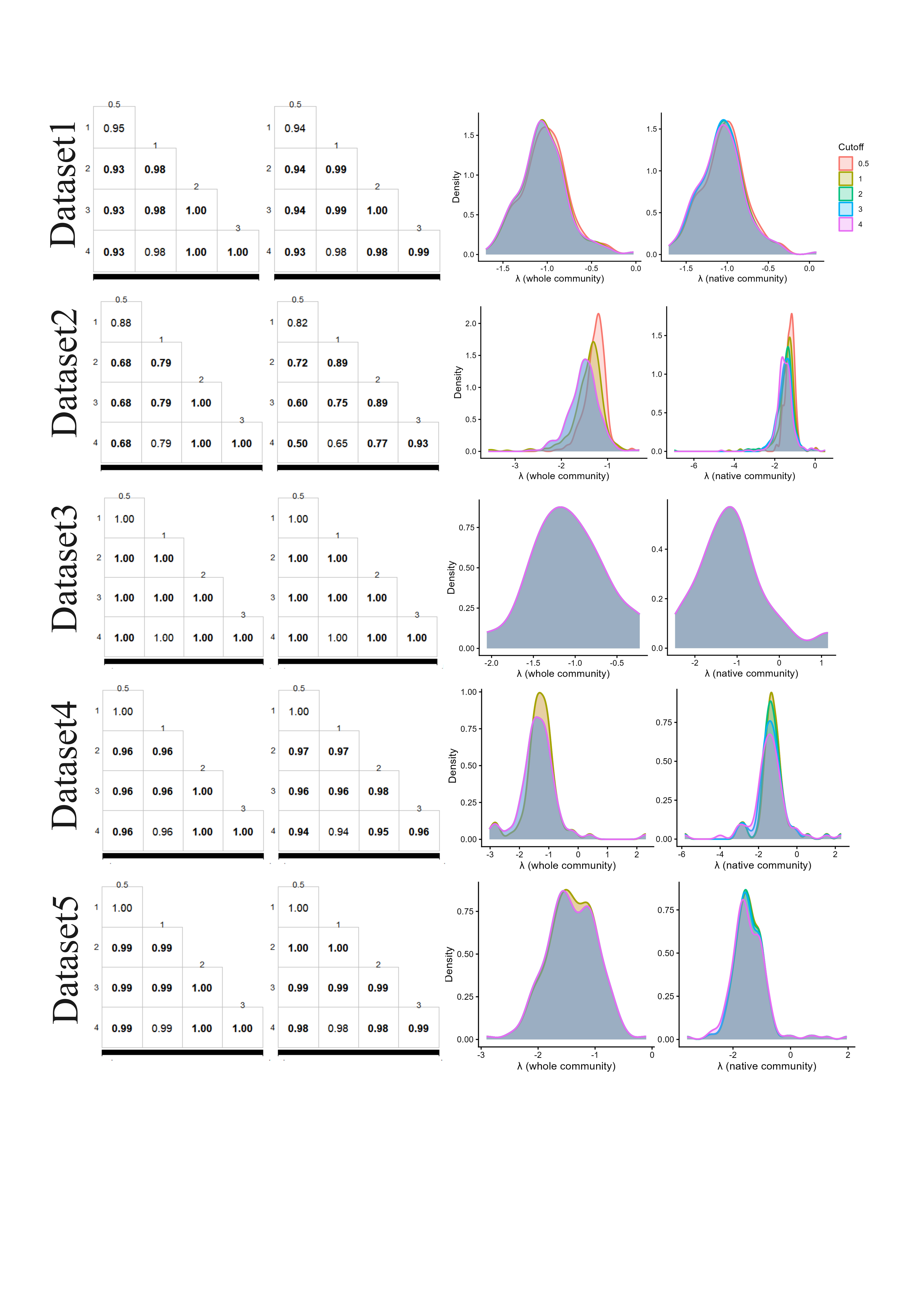

Supplement: Supplementary file 1 — Figure S1: LMM Model's diagnostic for the exponent of the whole community. [file GCB-32-e70884-s002.png]

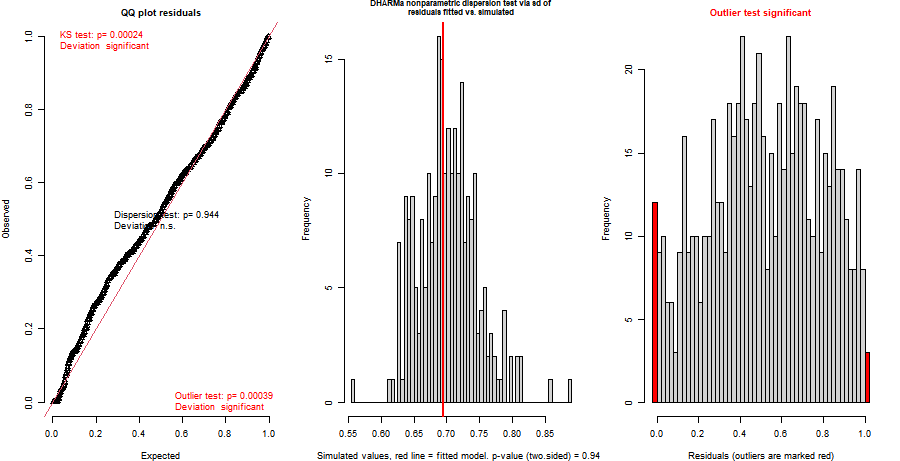

Supplement: Supplementary file 2 — Figure S2: LMM Model's diagnostic for total biomass of the whole community. [file GCB-32-e70884-s001.tiff]

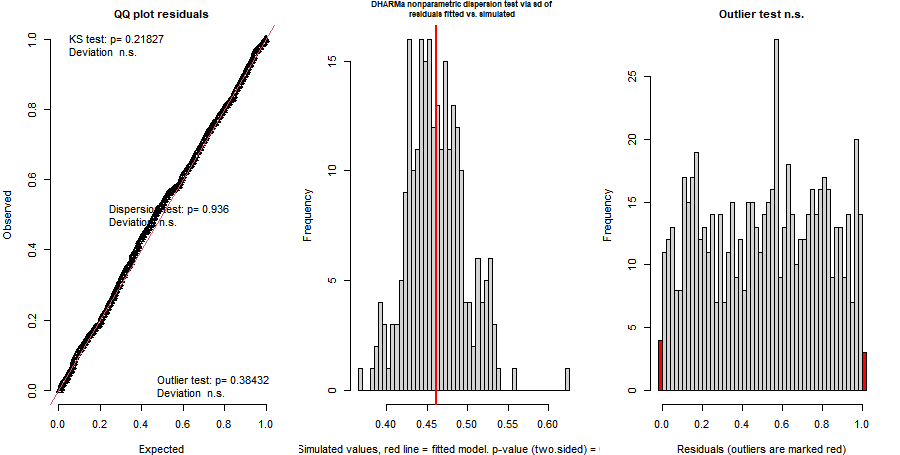

Supplement: Supplementary file 3 — Figure S3: LMM Model's diagnostic for the exponent of the native community. [file GCB-32-e70884-s004.tiff]

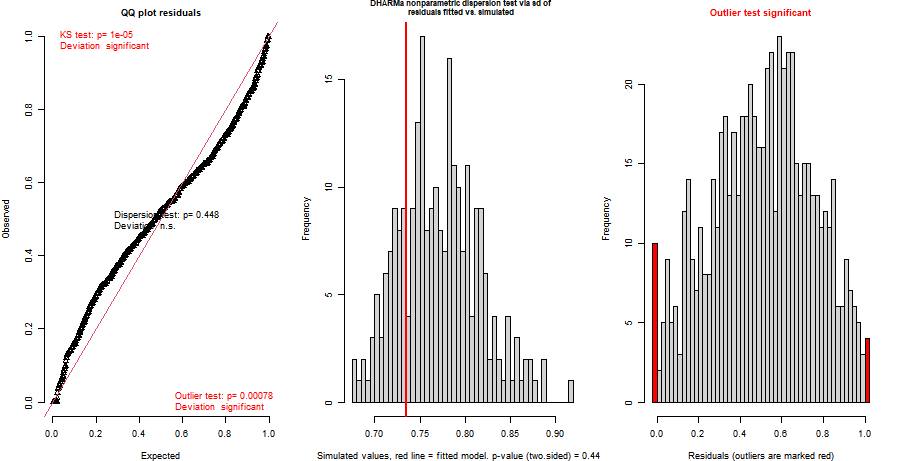

Supplement: Supplementary file 4 — Figure S4: LMM Model's diagnostic for total biomass of the native community. [file GCB-32-e70884-s008.tiff]

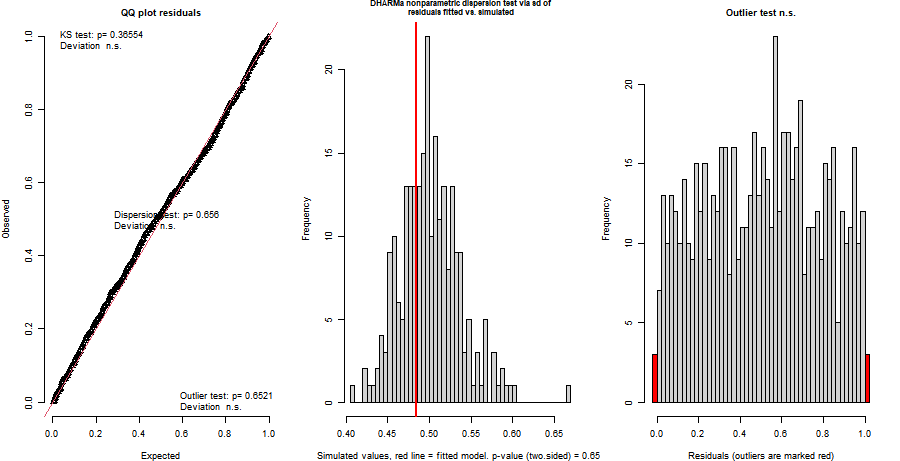

Supplement: Supplementary file 5 — Figure S5: Rank–frequency plots illustrating the bounded power‐law distribution of the size spectrum that was built. [file GCB-32-e70884-s006.tiff]

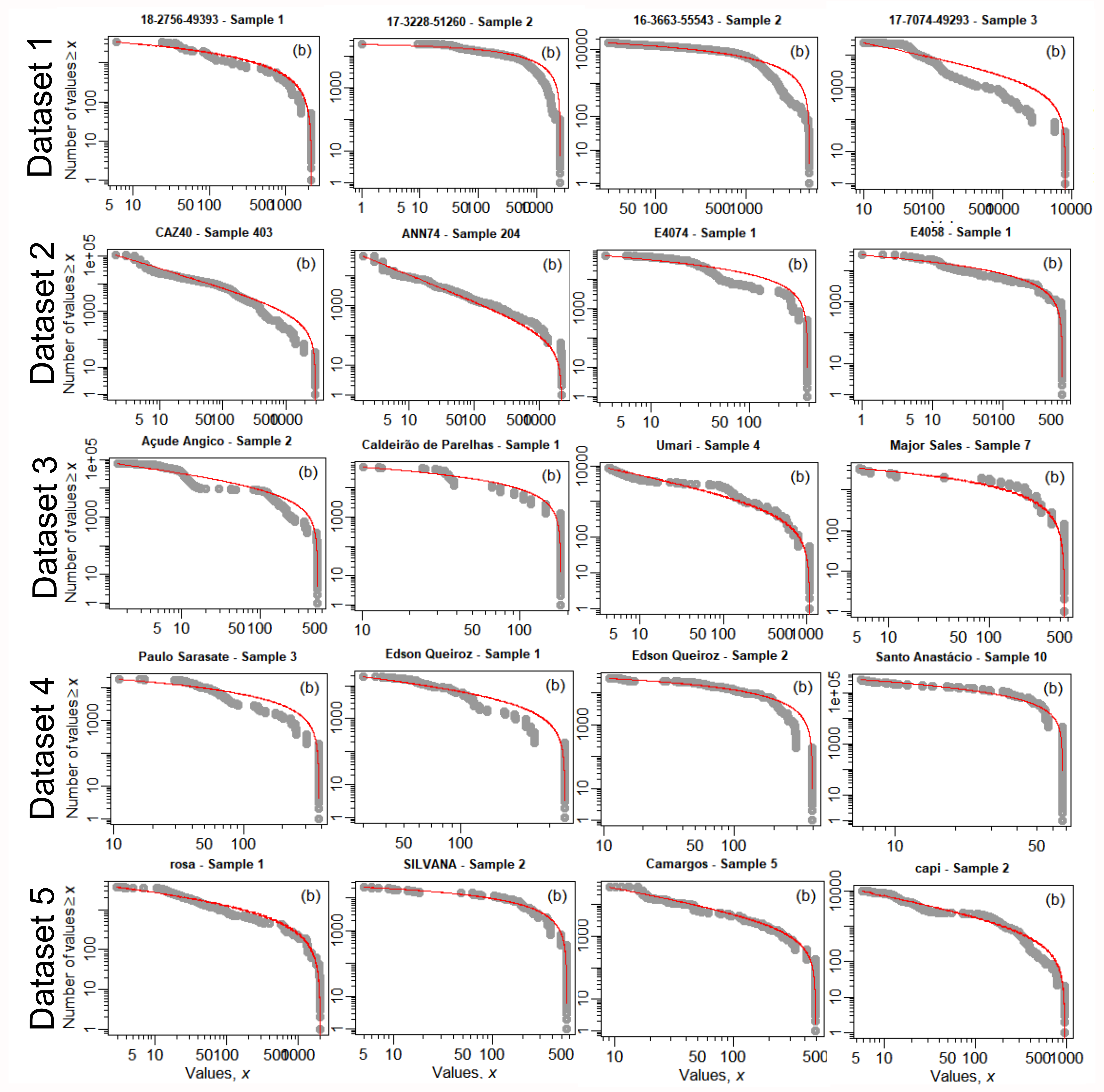

Supplement: Supplementary file 6 — Figure S6: Distribution of individual body biomass of native species within the size Class3 [4.42–13.14 g]. [file GCB-32-e70884-s005.png]

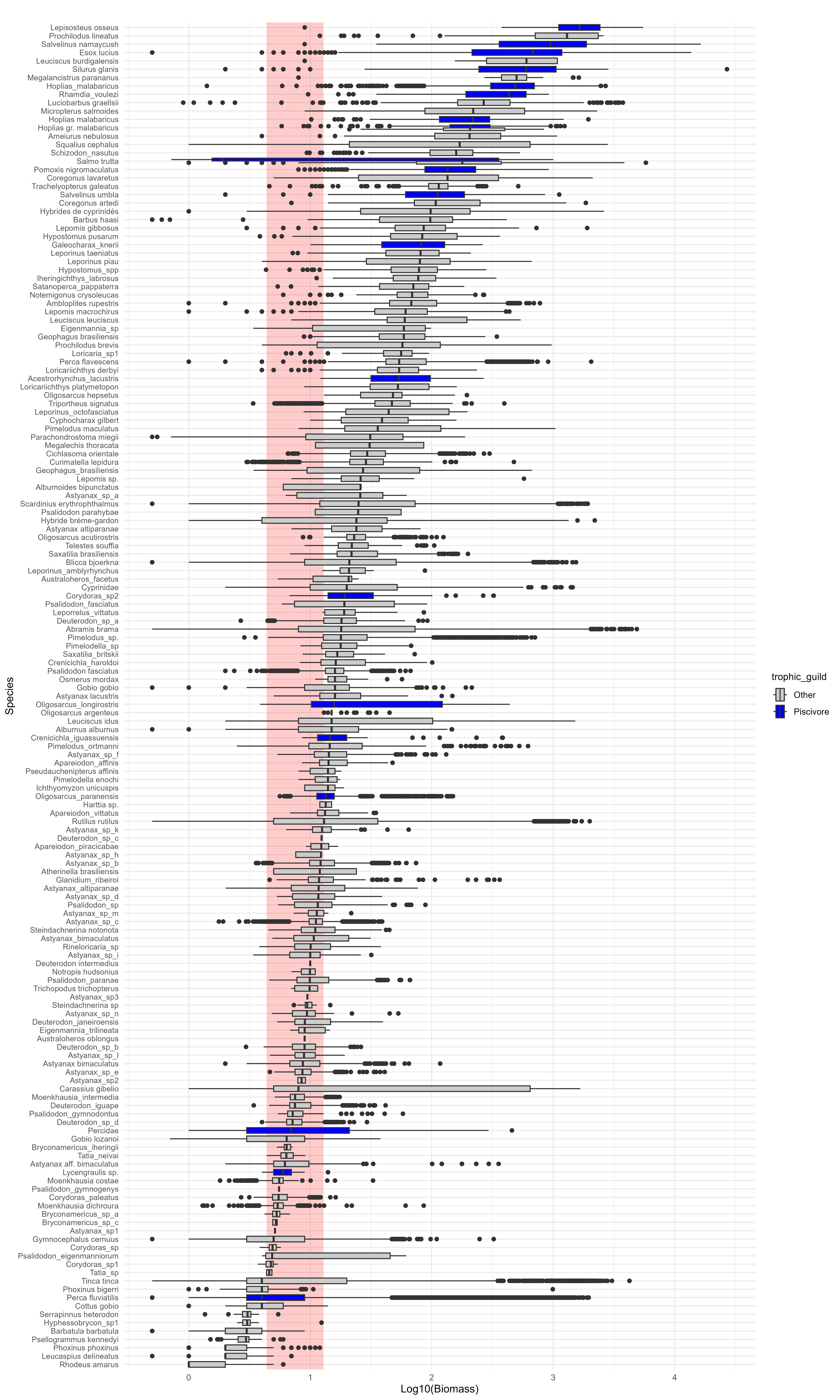

Supplement: Supplementary file 7 — Figure S7: Distribution of individual body biomass (log10‐transformed) for the 106 species occurring within their native range that fall within the size interval of Class 3 [4.42–13.14 g] of the native community (highlighted in red). This size class was found to be negatively correlated with both non‐native trophic groups. Boxplot colors indicate the trophic guild of the affected native species (blue = piscivores; grey = other trophic groups). [file GCB-32-e70884-s003.png]
