# Supplementary material for: Invasive Fishes Interact With Temperature to Reshape Community Size Structure Across Climatic Zones
Source: Glob Chang Biol. 2026 Apr 27;32:e70884. doi: 10.1111/gcb.70884 (PMC13112343; doi:10.1111/gcb.70884)
Supplement: Supplementary file 8 — Table S1: Overview of the datasets. Table S2: Descriptive statistics of abiotic and biotic covariates used in models. Table S3: Results of Robust linear model (RLM) for the exponent of whole community (native + NN). Table S4: Results of linear mixed model (LMM) for the total biomass of the whole community. Table S5: Results of RLM for the slope of native community. Table S6: Results of LMM for the total biomass of native community. Table S7: Results ofRLM for the exponent of whole community (native + NN) using the cutoff of 4 g for the individual body weight. Table S8: Results of RLM for slope of native community using the cutoff of 4 g for the individual body weight. [file GCB-32-e70884-s007.docx]

**Supporting Information**

**Invasive fishes interact with temperature to reshape community size structure across climatic zones**

Barbbara Silva Rocha¹, Ignasi Arranz²^,^³, Henrique C. Giacomini^4^, Daniel M. Perkins^5^, Gilberto Nepomuceno Salvador^6^, José Luís Costa Novaes^7^, Jorge Iván Sánchez-Botero^8^, Angelo Antonio Agostinho^9^, Paulo Santos Pompeu^10,^ Rosemberg Fernandes Menezes^11^, Silvia Yasmin Lustosa-Costa^12^, Telton Pedro A. Ramos^12, 13^, José Luiz Attayde^14^, Rodrigo Silva da Costa Goldbaum^15^, Christine Argillier^16^, Ronaldo César Gurgel-Lourenço^8^, Leonardo Mesquita Pinto^8^, Tiago Casarim Pessali^17^, María José Rodríguez–Pérez^18^, Victor Satoru Saito¹

Correspondence: Barbbara Silva Rocha (barbbararocha@ufscar.br; barbbararocha@gmail.com)

¹ Environmental Sciences Department, Federal University of São Carlos, São Carlos, São Paulo, Brazil

^2^ Instituto de Investigación en Cambio Global (IICG-URJC), Universidad Rey Juan Carlos, Tulipán s/n, 28933 Móstoles, España

^3^ Departamento de Biología y Geología, Física y Química Inorgánica, Universidad Rey Juan Carlos (URJC), Tulipán s/n, 28933 Móstoles, España

^4^ Ontario Ministry of Natural Resources, Aquatic Research and Monitoring Section, 2140 East Bank Drive, Peterborough ON K9L1Z8, Canada

^5^ Centre for Pollution Research and Policy, Brunel University London, Uxbridge, UB8 3PH, UK

^6^ Programa de Pós-Graduação em Ecologia, Conservação e Manejo da Vida Silvestre, Universidade Federal de Minas Gerais, Av. Pres. Antônio Carlos, 6627, Pampulha, Belo Horizonte, MG, Brasil

^7^ Biosciences Department, Federal Rural University of the Semi-Arid, Brazil

^8^ Laboratório de Ecologia Aquática e Conservação (LEAC), Departamento de Biologia, Universidade Federal do Ceará – Campus do Pici, Av. Mister Hull n/n, Fortaleza, 60440-900, Brazil

^9^ Programa de Pós-Graduação em Ecologia de Ambientes Aquáticos Continentais da Universidade Estadual de Maringá - 87020-900 Maringá - PR, Brazil

^10^ Departamento de Ecologia e Conservação, Universidade Federal de Lavras. Campus Universitário, Lavras, MG, 37203-202, Brasil

^11^ Universidade Federal da Paraíba (UFPB), Departamento de Fitotecnia e Ciências Ambientais, Centro de Ciências Agrárias, Rodovia PB-079, Km 12, 58397-000 Areia, PB, Brazil

^12^ Instituto Peixes da Caatinga, Rua Doutor Antônio Massa, 73, Jaguaribe, 58015-410 João Pessoa, PB, Brazil

^13^ Programa de Pós-Graduação em Sistemática e Evolução (UFRN), Departamento de Botânica e Zoologia,, Centro de Biociências, Universidade Federal do Rio Grande do Norte, Av. Senador Salgado Filho, 3000, Lagoa Nova, 59978-970 Natal, RN, Brazil

^14^ Departamento de Ecologia, Centro de Biociências, Universidade Federal do Rio Grande do Norte (UFRN), Natal, Rio Grande Do Norte, Brazil

^15^ Departamento de Biociências, Universidade Federal Rural do Semi-Árido, Av. Francisco Mota, 572 - Bairro Costa e Silva, Mossoró 59625-900, Rio Grande do Norte, Brazil

^16^ INRAE, Aix Marseille University, UMR RECOVER, Aix-en-Provence, France

^17^ Universidade Federal de Minas Gerais, Av. Pres. Antônio Carlos, 6627, Pampulha, Belo Horizonte, MG, Brasil

^18^ Confederación Hidrográfica del Ebro Paseo Sagasta, Zaragoza, Spain

This document contains the following:

**1. Supporting Methods**

Imputation protocol

**2. Supporting Tables**

**Table S1 -** Overview of the datasets.

**Table S2 -** Descriptive statistics of abiotic and biotic covariates used in models.

**Table S3 –** Results of Robust linear model (RLM) for the exponent of whole community (native + NN).

**Table S4** – Results of linear mixed model (LMM) for total biomass of the whole community.

**Table S5 –** Results of RLM for slope of native community.

**Table S6 –** Results of LMM for total biomass of native community.

**Table S7 –** Results of Robust linear model (RLM) for the exponent of whole community (native + NN) using the cutoff of 4g for the individual body weight.

**Table S8 –** Results of RLM for slope of native community using the cutoff of 4g for the individual body weight.

**3. Supporting Figures**

**Figure S1 –** LMM Model’s diagnostic for the exponent of the whole community.

**Figure S2 –** LMM Model’s diagnostic for total biomass of the whole community.

**Figure S3 –** LMM Model’s diagnostic for the exponent of the native community.

**Figure S4 –** LMM Model’s diagnostic for total biomass of the native community.

**Figure S5** – Rank–frequency plots illustrating the bounded power-law distribution of the size spectrum that was built.

**Figure S6:** Distribution of individual body biomass of native species within the size class3 [4.42–13.14 g].

**1. Supporting Methods**

*Body mass data imputation*

To deal with missing data on individual body mass, we applied a standardized imputation procedure across datasets. When total length information was available, body mass was estimated using species-specific length–weight relationships (LWR; *W = a L^b^*) derived from individuals sampled in the same region. For species with few individuals, the parameters *a* and *b* were complemented with published values available in literature [(Froese & Pauly, 2021)](https://www.zotero.org/google-docs/?PFxcof), giving preference to studies conducted in nearby or ecologically similar systems.

When both body mass and length data were missing for some individuals, we constructed right-skewed (log-normal) distributions based on individuals of the same species sampled at the same site and used these distributions to estimate the missing values. We included only sampling events in which no more than 5% of the total individuals sampled had missing values for both body length and mass, to ensure sufficient sample size for reliable imputation and to preserve the quality of the sampled data. The imputed values were visually inspected for outliers and consistency within species.

**3. Supporting Tables**

**Table S1.** Overview of the datasets used in this study. The column ID refers to the dataset identifier used in the statistical models. “Years” indicates the range of years during which the surveys were conducted. “N” represents the total number of surveys. “M_sizes” shows the minimum and maximum mesh sizes (in cm) of the gillnet gangs used in the sampling protocol. Protocol indicates the official name of the sampling protocol, if applicable. The “Reference” provides methodological details on the sampling applied.

| ID | Region | Country | Years | N | M_sizes | Protocol | Reference |
| --- | --- | --- | --- | --- | --- | --- | --- |
| 1 | Temperate | Canada | 2008 to 2024 | 118 | 3.8-12.7 | “North American”**^*^** | [Arranz et al. (2022)](https://www.zotero.org/google-docs/?tojFpW) |
| 2 | Temperate | France;  Spain | 2005 to 2023 | 381 | 0.5-5.5 | “CEN”**^**^** | [Rocha et al. (2024)](https://www.zotero.org/google-docs/?kNqks1) |
| 3 | Tropical | Brazil | 2010 to 2022 | 66 | 1.2-12 | - | [De Oliveira et al., 2021; Ramos et al., 2021; Rocha et al., 2021](https://www.zotero.org/google-docs/?aL8Jtg) |
| 4 | Tropical | Brazil | 2009 to 2019 | 19 | 2.5-12 | - | [Bezerra et al. (2018); Gurgel-Lourenço et al. (2013); Sánchez-Botero et al. (2014)](https://www.zotero.org/google-docs/?BKByF9) |
| 5 | Tropical/Sub-tropical | Brazil | 2001 to 2024 | 146 | 2.4-16 | - | [Muniz et al. (2020); Salvador et al. (2022); Silva-Sene et al. (2023)](https://www.zotero.org/google-docs/?0CVxVe) |

**^*^** Sandstrom, S.J., M.R. Rawson and N.P. Lester. 2015. Manual of Instructions for Inland Lakes Broad-scale Monitoring; Part 1 Netting Survey; version 2015.1. Ontario Ministry of Natural Resources and Forestry, Science and Research Branch, Biodiversity and Monitoring Section, Peterborough,ON. 35 pp. + appends.

**^**^** CEN, 2015. Water Quality—Sampling of Fish with Multi-mesh Gillnets (EN 14757:2015).

**Table S2**: Descriptive statistics (minimum, maximum, mean, and standard deviation) of abiotic and biotic covariates included in the models.

| Variable | Min | Max | Mean | sd |
| --- | --- | --- | --- | --- |
| Lake_area (km²) | 0.004 | 4496.7 | 53.20 | 356.08 |
| Max_depth (m) | 1.10 | 309.7 | 30 | 29.77 |
| TotP (µg/L) | 1.90 | 2012.00 | 69.20 | 156.18 |
| MeanAnTemp (°C) | -0.04 | 29.22 | 14.40 | 7.46 |
| Precip | -1.96 | 5.30 | 0.04 | 1.36 |
| sp_rich | 2 | 37 | 10 | 4.88 |

**Table S3**: Results of the robust linear mixed model (RLM) assessing the effect of invasion pressure (PCA_inv) and covariates on the size spectrum exponent of the entire community (native + NN species). Estimates. Standard Error. t-values and p-values are shown. Significant *p*-values are in bold. R2 adj = 0.24.

| **Predictor** | **Estimate** | **Std. Error** | **t-value** | **p-value** |
| --- | --- | --- | --- | --- |
| (Intercept) | 0.66 | 0.131 | 5.028 | **<0.001** |
| PCA_inv | 0.164 | 0.039 | 4.271 | **<0.001** |
| Temp | 0.038 | 0.074 | 0.51 | 0.61 |
| Dataset2 | -0.658 | 0.146 | -4.508 | **<0.001** |
| Dataset3 | -0.584 | 0.313 | -1.867 | 0.062 |
| Dataset4 | -0.53 | 0.284 | -1.862 | **0.063** |
| Dataset5 | -0.904 | 0.215 | -4.199 | **<0.001** |
| typeReservoir | -0.144 | 0.106 | -1.359 | 0.175 |
| Precip | 0.017 | 0.033 | 0.525 | 0.599 |
| TotP | 0.052 | 0.051 | 1.03 | 0.303 |
| sp_rich | -0.033 | 0.062 | -0.539 | 0.59 |
| area_km2 | -0.156 | 0.057 | -2.71 | **0.007** |
| max_depth | 0.142 | 0.039 | 3.643 | **<0.001** |
| PCA_inv:Temp | 0.246 | 0.044 | 5.545 | **<0.001** |

**Table S4**: Results of the linear mixed-effects model (LMM) testing the effect of invasion pressure (PCA_inv) and covariates on total biomass of the entire community (native + NN species). Estimates. standard errors. 95% confidence intervals. degrees of freedom. t-values. and p-values are shown. Significant *p*-values are in bold. R2c = 0.78. R2m = 0.54.

| **Predictor** | **Estimate** | **Std. Error** | **df** | **t-value** | **p-value** |
| --- | --- | --- | --- | --- | --- |
| (Intercept) | 0.468 | 0.137 | 447.697 | 3.415 | **<0.001** |
| PCA_inv | 0.071 | 0.038 | 578.701 | 1.896 | 0.058 |
| Temp | 0.033 | 0.073 | 461.494 | 0.425 | 0.671 |
| Lake_type(Reservoir) | 0.153 | 0.111 | 361.062 | 1.356 | 0.176 |
| dataset4 | -0.834 | 0.372 | 259.375 | -2.24 | **0.026** |
| dataset2 | -0.146 | 0.159 | 392.759 | -0.9 | 0.369 |
| dataset5 | -1.812 | 0.215 | 377.693 | -8.39 | **<0.001** |
| dataset3 | -1.684 | 0.298 | 359.679 | -5.651 | **<0.001** |
| TotP | 0.180 | 0.036 | 651.689 | 5.045 | **<0.001** |
| sp_rich | 0.120 | 0.04 | 641.661 | 3.144 | **0.002** |
| Precip | 0.009 | 0.034 | 649.631 | 0.268 | 0.788 |
| area_km2 | -0.089 | 0.051 | 411.961 | -1.74 | 0.083 |
| max_depth | -0.13 | 0.035 | 588.003 | -3.654 | **<0.001** |
| PCA_inv:Temp | 0.126 | 0.038 | 624.305 | 3.288 | **0.001** |

**Table S5.** Results of the robust linear model (RLM) assessing the effects of invasion pressure from piscivorous non-native species (“Pisc_NN”) and from non-native species at lower trophic levels (“LTL_NN”). along with covariates on the size spectrum exponent of native fish communities. Estimates. standard errors. 95% confidence intervals. t-values. and p-values are shown. Significant *p*-values are in bold. R2 adj = 0.27

| **Predictor** | **Estimate** | **Std. Error** | **t-value** | **p-value** |
| --- | --- | --- | --- | --- |
| (Intercept) | 0.488 | 0.111 | 4.413 | **<0.001** |
| Others_NN | 0.118 | 0.031 | 3.852 | **<0.001** |
| Temp | 0.005 | 0.085 | 0.056 | 0.955 |
| Pisc_NN | 0.083 | 0.033 | 2.48 | **0.013** |
| dataset4 | -0.671 | 0.421 | -1.594 | 0.111 |
| dataset2 | -0.575 | 0.112 | -5.115 | **<0.001** |
| dataset5 | -0.992 | 0.18 | -5.52 | **<0.001** |
| dataset3 | -0.622 | 0.238 | -2.613 | **0.009** |
| typeReservoir | 0.066 | 0.076 | 0.877 | 0.381 |
| Precip | -0.009 | 0.026 | -0.349 | 0.727 |
| TotP | 0.034 | 0.04 | 0.844 | 0.399 |
| sp_rich | -0.011 | 0.046 | -0.241 | 0.81 |
| area_km2 | -0.119 | 0.044 | -2.695 | **0.007** |
| max_depth | 0.095 | 0.028 | 3.384 | **0.001** |
| Others_NN:Temp | 0.03 | 0.047 | 0.625 | 0.532 |
| Temp x Pisc_NN | 0.15 | 0,063 | 2,364 | **0.018** |

**Table S6**: Results of the robust linear mixed-effects model (LMM) testing the effect of invasion pressure of piscivores (“NN_Pisc”) and those from lower trophic levels (“NN_Others”) and covariates on total biomass of native communities. Estimates. standard errors. 95% confidence intervals. degrees of freedom. t-values. and p-values are shown. Significant *p*-values are in bold. R2c = 0.80. R2m = 0.50.

| **Predictor** | **Estimate** | **Std. Error** | **df** | **t-value** | **p-value** |
| --- | --- | --- | --- | --- | --- |
| (Intercept) | 0.531 | 0.129 | 416.455 | 4.104 | **<0.001** |
| Others_NN | -0.119 | 0.033 | 558.098 | -3.594 | **<0.001** |
| Temp | 0.006 | 0.073 | 461.808 | 0.084 | 0.933 |
| Pisc_NN | -0.097 | 0.036 | 573.718 | -2.699 | **0.007** |
| dataset4 | -0.695 | 0.398 | 232.93 | -1.749 | 0.082 |
| dataset2 | -0.204 | 0.15 | 372.802 | -1.355 | 0.176 |
| dataset5 | -1.629 | 0.212 | 363.336 | -7.671 | **<0.001** |
| dataset3 | -1.456 | 0.293 | 352.15 | -4.979 | **<0.001** |
| typeReservoir | 0.097 | 0.115 | 367.198 | 0.843 | 0.4 |
| TotP | 0.2 | 0.036 | 621.922 | 5.505 | **<0.001** |
| sp_rich | 0.183 | 0.04 | 616.015 | 4.622 | **<0.001** |
| Precip | -0.001 | 0.033 | 615.077 | -0.033 | 0.974 |
| area_km2 | -0.096 | 0.051 | 401.759 | -1.875 | 0.062 |
| max_depth | -0.105 | 0.035 | 562.749 | -3.006 | **0.003** |
| NN_others: Temp | 0.094 | 0.042 | 624.653 | 2.222 | **0.027** |
| Temp:NN_pisc | 0.041 | 0.046 | 614.676 | 0.896 | 0.371 |

**Table S7**: Results of the robust linear mixed model (RLM) assessing the effect of invasion pressure (PCA_inv) and covariates on the size spectrum exponent of the entire community (native + NN species) using the cutoff of 4g for the individual body weight. Estimates. Standard Error. t-values and p-values are shown. Significant *p*-values are in bold. R2 adj = 0.35

| **Predictor** | **Estimate** | **Std. Error** | **t-value** | **p-value** |
| --- | --- | --- | --- | --- |
| (Intercept) | 0.862 | 0.117 | 7.34 | **<0.001** |
| PCA_inv | 0.187 | 0.041 | 4.543 | **<0.001** |
| Temp | -0.049 | 0.058 | -0.833 | 0.405 |
| dataset4 | -0.5 | 0.255 | -1.958 | 0.051 |
| dataset2 | -1.121 | 0.135 | -8.309 | **<0.001** |
| dataset5 | -0.731 | 0.178 | -4.1 | **<0.001** |
| dataset3 | -0.545 | 0.247 | -2.205 | **0.028** |
| typeReservoir | -0.152 | 0.109 | -1.391 | 0.165 |
| Precip | -0.085 | 0.036 | -2.393 | 0.017 |
| TotP | -0.012 | 0.049 | -0.237 | 0.813 |
| sp_rich | -0.113 | 0.06 | -1.883 | **0.06** |
| area_km2 | -0.132 | 0.053 | -2.514 | 0.012 |
| max_depth | 0.16 | 0.04 | 3.977 | **<0.001** |
| PCA_inv:Temp | 0.20 | 0.039 | 4.916 | **<0.001** |

**Table S8.** Results of the robust linear model (RLM) assessing the effects of invasion pressure from piscivorous non-native species (“Pisc_NN”) and from non-native species at lower trophic levels (“LTL_NN”), along with covariates, on the size spectrum exponent of native fish communities. Estimates, standard errors, 95% confidence intervals, t-values, and p-values are shown. Significant p-values are in bold. R2 adj = 0.34.

| **Predictor** | **Estimate** | **Std. Error** | **t-value** | **p-value** | |
| --- | --- | --- | --- | --- | --- |
| (Intercept) | 0.595 | 0.087 | 6.837 | **<0.001** |  |
| Others_NN | 0.142 | 0.027 | 5.188 | **<0.001** |  |
| Temp | -0.021 | 0.071 | -0.3 | 0.764 |  |
| Pisc_NN | 0.121 | 0.031 | 3.951 | **<0.001** |  |
| dataset4 | -0.464 | 0.335 | -1.387 | 0.166 |  |
| dataset2 | -0.773 | 0.095 | -8.146 | **<0.001** |  |
| dataset5 | -0.61 | 0.14 | -4.366 | **<0.001** |  |
| dataset3 | -0.44 | 0.195 | -2.261 | **0.024** |  |
| typeReservoir | -0.086 | 0.077 | -1.12 | 0.263 |  |
| Precip | -0.046 | 0.026 | -1.761 | 0.079 |  |
| TotP | -0.012 | 0.034 | -0.368 | 0.713 |  |
| sp_rich | -0.096 | 0.042 | -2.298 | **0.022** |  |
| area_km2 | -0.098 | 0.038 | -2.589 | **0.01** |  |
| max_depth | 0.106 | 0.027 | 3.997 | **<0.001** |  |
| Others_NN:Temp | 0.012 | 0.042 | 0.293 | 0.77 |  |
| Temp x Pisc_NN | 0.131 | 0.049 | 2.669 | **0.008** |  |


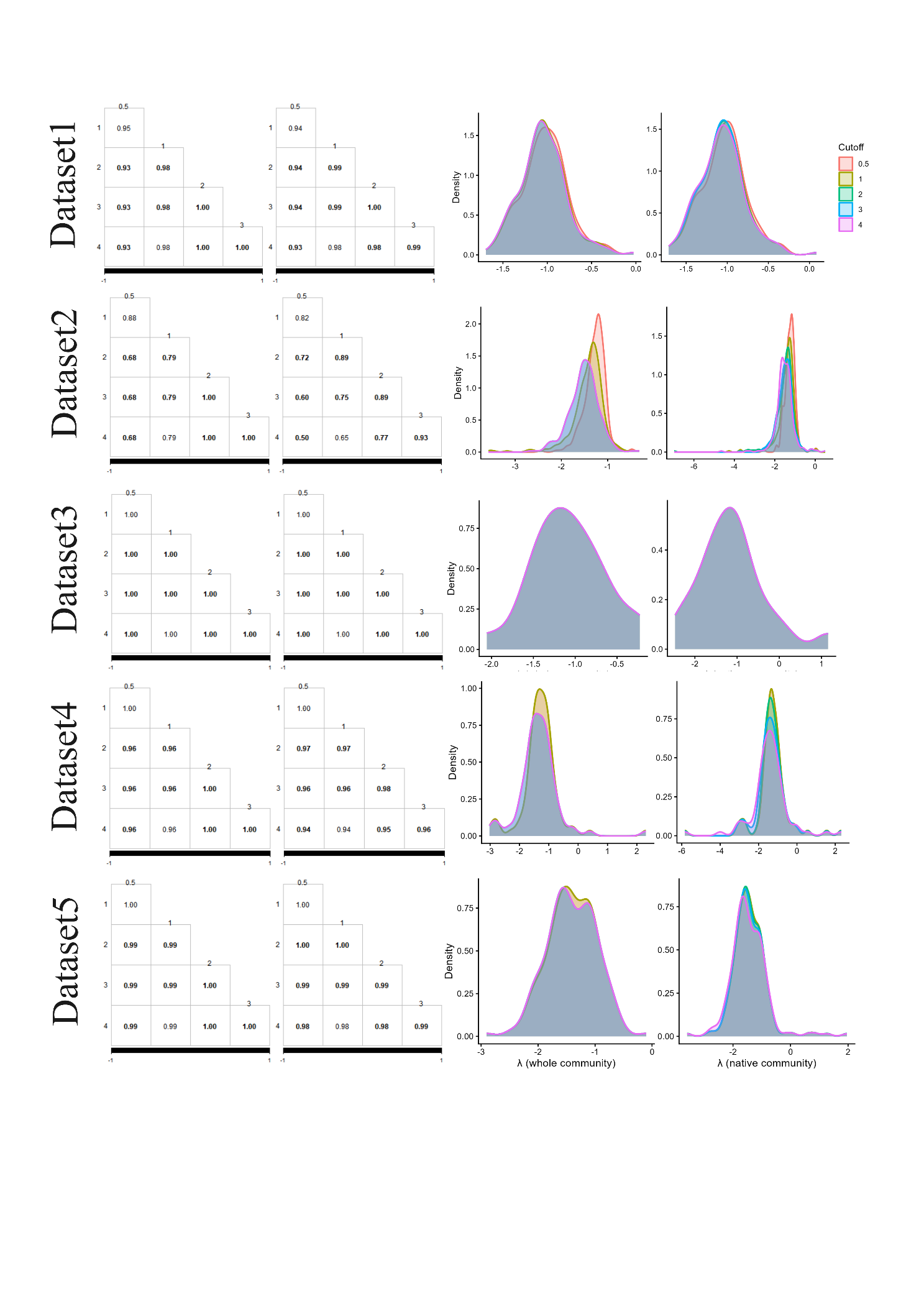


**Figure S1.** Sensitivity of size spectrum exponent (λ) estimates to different cutoff values across the five datasets. Correlograms (left panels) show pairwise correlations of λ estimated using different cutoff values (0.5, 1, 2, 3, and 4) for the whole community (all species) and for native species only, respectively. Density plots (right panels) illustrate the distribution of λ values obtained under each cutoff for both community definitions. Overall, high correlations in bold indicate strong and significant (p < 0.05) consistency in λ estimates across cutoff thresholds. In contrast, Dataset 2 (“CEN” protocol) showed comparatively lower correlations (<90%) among cutoff values, indicating that λ estimates in this dataset are more sensitive to the choice of cutoff.


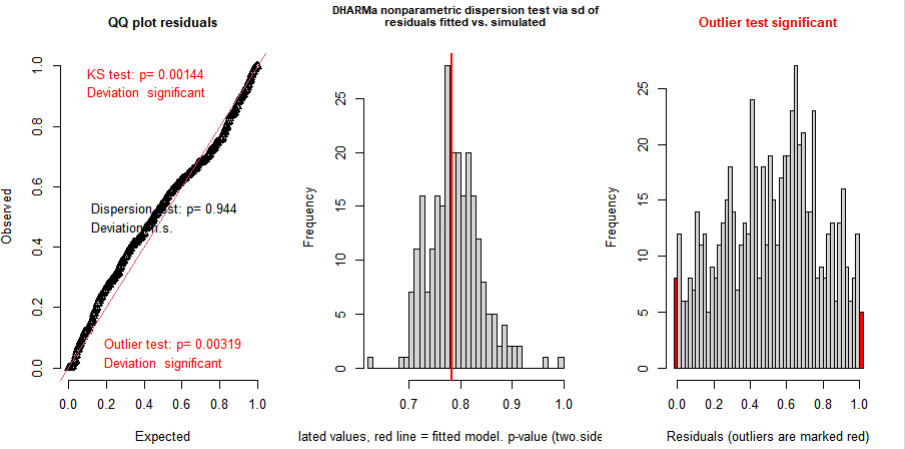


**Figure S2.** Diagnostic test of model residuals with the response variable “exponent(all)”, performed using the DHARMa package.


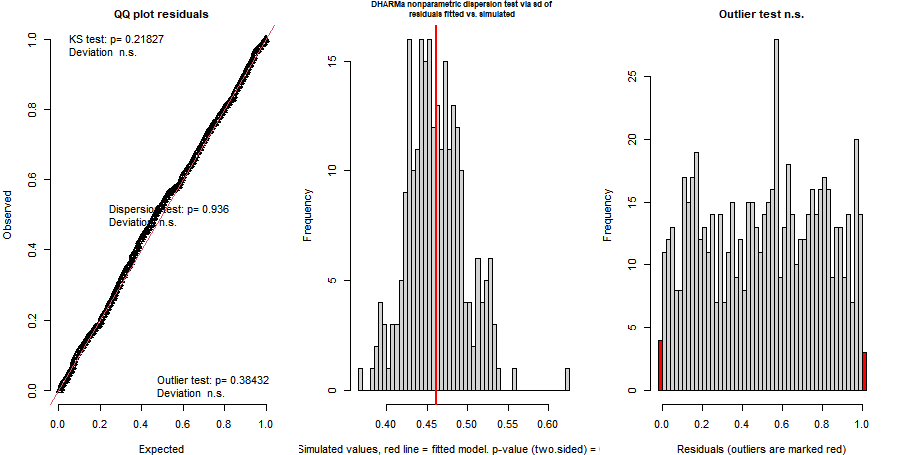


**Figure S3.** Diagnostic test of model residuals with the response variable “tot_biomass(all)”, performed using the DHARMa package.


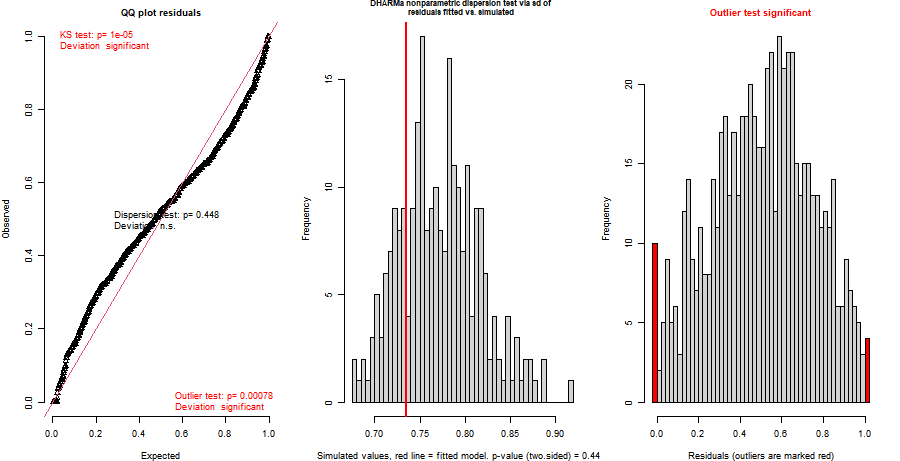
**Figure S4.** Diagnostic test of model residuals with the response variable “exponent(native)”, performed using the DHARMa package.


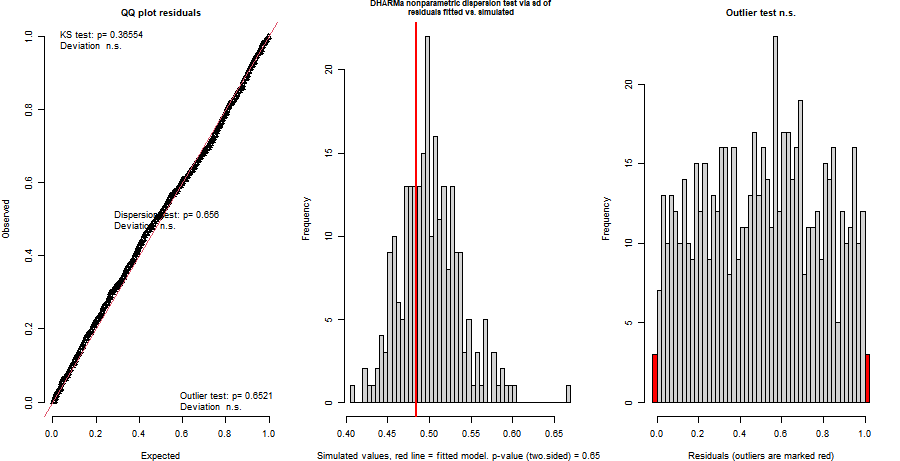
w

**Figure S5.** Diagnostic test of model residuals with the response variable “tot_biomass(native)”, performed using the DHARMa package.


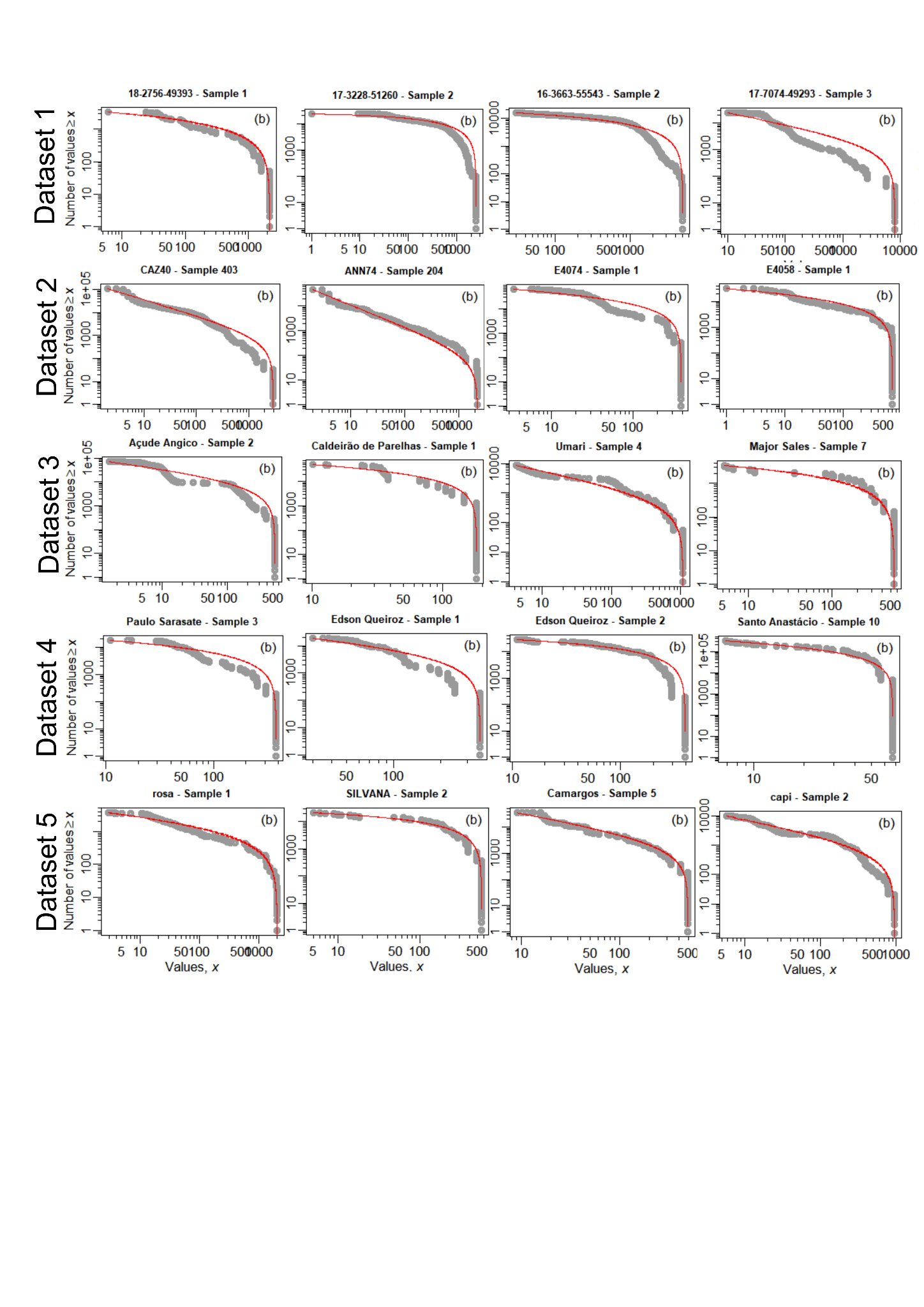


**Figure S6**: A subset of rank–frequency plots illustrating that the bounded power-law distribution (red line) provides a good fit to the empirical data. Each plot represents a community (native + NN) considering the different datasets used in the study (see Table S1 for dataset ID). The lake name and sampling event number are shown above each plot.


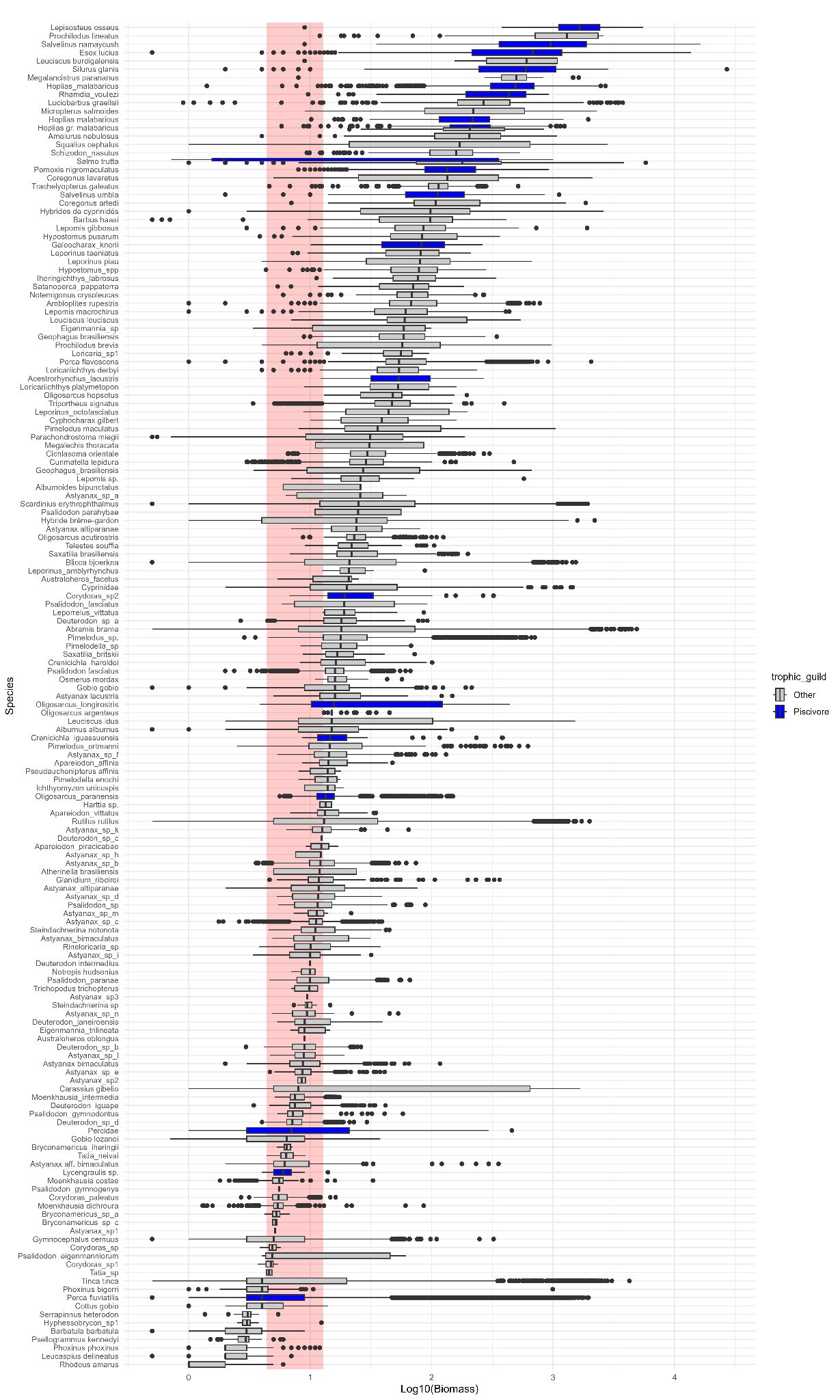


**Figure S7:** Distribution of individual body biomass (log10-transformed) for the 106 species occurring within their native range that fall within the size interval of Class 3 [4.42–13.14 g] of the native community (highlighted in red). This size class was found to be negatively correlated with both non-native trophic groups. Boxplot colors indicate the trophic guild of the affected native species (blue = piscivores; grey = other trophic groups).

**Reference**

[Arranz, I., Fournier, B., Lester, N. P., Shuter, B. J., & Peres‐Neto, P. R. (2022). Species compositions mediate biomass conservation: The case of lake fish communities. Ecology, 103(3), e3608. https://doi.org/10.1002/ecy.3608](https://www.zotero.org/google-docs/?dkZrXA)

[Bezerra, L. A. V., Angelini, R., Vitule, J. R. S., Coll, M., & Sánchez-Botero, J. I. (2018). Food web changes associated with drought and invasive species in a tropical semiarid reservoir. Hydrobiologia, 817(1), Artigo 1.](https://www.zotero.org/google-docs/?dkZrXA)

[De Oliveira, J. C. D., De Oliveira, J. F., Marques, A. D. O., Peretti, D., Da Costa, R. S., & Novaes, J. L. C. (2021). Trophic ecology of detritivorous fish along a reservoir cascade in a tropical semi‐arid region. Ecology of Freshwater Fish, 30(2), 234–243. https://doi.org/10.1111/eff.12579](https://www.zotero.org/google-docs/?dkZrXA)

[Froese, R., & Pauly, D. (2021). FishBase. World wide web electronic publication. http://www. fishbase. org.](https://www.zotero.org/google-docs/?dkZrXA)

[Gurgel-Lourenço, R. C., Rodrigues-Filho, C. A. de S., Angelini, R., Garcez, D. S., & Sánchez-Botero, J. I. (2015). On the relation amongst limnological factors and fish abundance in reservoirs at semiarid region. Acta Limnologica Brasiliensia, 27(1), Artigo 1. https://doi.org/10.1590/S2179-975X2414](https://www.zotero.org/google-docs/?dkZrXA)

[Muniz, C. M., Ganassin, M. J. M., Agostinho, A. A., & Gomes, L. C. (2020). Spatial and environmental factors predict the composition of non-native fish assemblages in Neotropical reservoirs. Biological Invasions, 22(2), Artigo 2.](https://www.zotero.org/google-docs/?dkZrXA)

[Ramos, T. P. A., Lustosa-Costa, S. Y., Lima, R. M. O., Barbosa, J. E. de L., & Menezes, R. F. (2021). First record of Moenkhausia costae (Steindachner 1907) in the Paraíba do Norte basin after the São Francisco River diversion. Biota Neotropica, 21(2), e20201049.](https://www.zotero.org/google-docs/?dkZrXA)

[Rocha, B. S., García-Berthou, E., Novaes, J. L. C., Bini, L. M., & Cianciaruso, M. V. (2021). Interspecific synchrony is related to body-length similarity in a fish community under prolonged drought conditions. Science of the Total Environment, 781, 146721.](https://www.zotero.org/google-docs/?dkZrXA)

[Rocha, B. S., Jamoneau, A., Logez, M., Laplace-Treyture, C., Reynaud, N., & Argillier, C. (2024). Measuring biodiversity vulnerability in French lakes–The IVCLA index. Science of The Total Environment, 908, 168205.](https://www.zotero.org/google-docs/?dkZrXA)

[Salvador, G. N., Montag, L. F. A., Hughes, R. M., Almeida, S. M., Prudente, B. S., Pessali, T. C., Barroso, T. A., Cianciaruso, M. V., Ligeiro, R., Juen, L., & Carlucci, M. B. (2022). Influences of multiple anthropogenic disturbances coupled with a tailings dam rupture on spatiotemporal variation in fish assemblages of a tropical river. Freshwater Biology, 67(10), 1708–1724. https://doi.org/10.1111/fwb.13967](https://www.zotero.org/google-docs/?dkZrXA)

[Sánchez-Botero, J. I., Reis, V. C. dos, Chaves, F. D. do N., & Garcez, D. S. (2014). Fish assemblage of the Santo Anastácio reservoir (Ceará state, Brazil). https://repositorio.ufc.br/handle/riufc/68504](https://www.zotero.org/google-docs/?dkZrXA)

[Silva-Sene, A. M., Fontes, R. C., Prado, I. G., & Pompeu, P. S. (2023). Migratory fishes from rivers to reservoirs: Seasonal and longitudinal perspectives. Zoologia (Curitiba), 40, e22044.](https://www.zotero.org/google-docs/?dkZrXA)
